# Aneuploidy Is Associated with Azole Resistance in Aspergillus fumigatus

**DOI:** 10.1128/aac.01253-22

**Published:** 2023-03-28

**Authors:** Aiah Khateb, Sara Gago, Michael Bromley, Malcom Richardson, Paul Bowyer

**Affiliations:** a Manchester Fungal Infection Group, Division of Evolution Genomics, School of Biological Sciences, Faculty of Biology, Medicine and Health, The University of Manchester, Core Technology Facility, Manchester, United Kingdom; b Department of Medical Laboratory Technology, Collage of Applied Medical Science, Taibah University, Medina, Saudi Arabia; c Special Infectious Agents Unit BSL-3, King Fahd Medical Research Center, King Abdulaziz University, Jeddah, Saudi Arabia; d Mycology Reference Centre Manchester, ECMM Centre of Excellence, Manchester University NHS Foundation Trust, Manchester, United Kingdom

**Keywords:** Aspergillus fumigatus, Mycology, antifungal resistance, azole, genome analysis, genome organization

## Abstract

Azole resistance in Aspergillus fumigatus is on the rise. Nontarget-mediated mechanisms are a common cause of azole resistance in chronic pulmonary aspergillosis (CPA). Here, we investigate resistance mechanisms using whole-genome sequencing. Sixteen azole-resistant A. fumigatus isolates from CPA were sequenced to assess genome rearrangements. Seven out of 16 CPA isolates showed genomic duplications compared to zero out of 18 invasive isolates. Duplication of regions, including *cyp*51A, increased gene expression. Our results suggest aneuploidy as an azole resistance mechanism in CPA.

## INTRODUCTION

Aspergillus fumigatus is the predominant causative agent of aspergillosis, a disease that affects over 10 million people globally and is annually responsible for >650,000 deaths (1, 2). There are few effective drugs for aspergillosis therapy, and only three classes of drugs are currently used in clinical practice with the azole classes representing the frontline therapy for these pulmonary infections. Azole therapy for aspergillosis was introduced in the 1970s with itraconazole and now involves progressively more potent and tolerable compounds. Current therapy is based on itraconazole, voriconazole, posaconazole, and isavuconazole; however, azole resistance and therapeutic failure are emerging.

The course of aspergillosis can either be rapid, lasting a few weeks or months in invasive infections, or protracted, lasting many years in the case of chronic infections. This disparity between duration of infection, and hence treatment, may lead to differences in the types of resistance mechanisms observed in each manifestation of the disease. Such differences may also arise from the different modes of infection, with hyphae in invasive disease rapidly ramifying through tissue and formation of fungal masses (aspergillomas) in expanding cavities in chronic infections. Estimated mortality for chronic disease is about 400,000 per year (2).

Resistance to azoles is an acknowledged global problem. Increasing azole resistance has been well documented in recent years (3–5). Many isolates from invasive aspergillosis carry alterations in the *cyp51*A target gene but many isolates are resistant due to unknown mechanisms and this proportion of non-*cyp51*A mediated resistance appears to be higher in longer term forms of infection such as chronic pulmonary aspergillosis (CPA).

Aneuploidy in fungi is a well-studied phenomenon (6, 7). Recent work in Candida albicans and Cryptococcus neoformans suggests that aneuploidy involving repeated chromosomal regions containing key azole resistance genes is a possible resistance mechanism in fungal pathogens of humans (8, 9).

Here, we observed aneuploidy in non-*cyp51A* azole resistant A. fumigatus isolates from clinically defined CPA patients. The aneuploidy involves duplication of chromosome regions containing genes relevant to the ergosterol biosynthesis pathway or other genes previously identified in azole resistance, either in laboratory screens or in wild isolates. This is the first observation of clinically relevant aneuploidy related to drug resistance in A. fumigatus.

**FIG 1:**
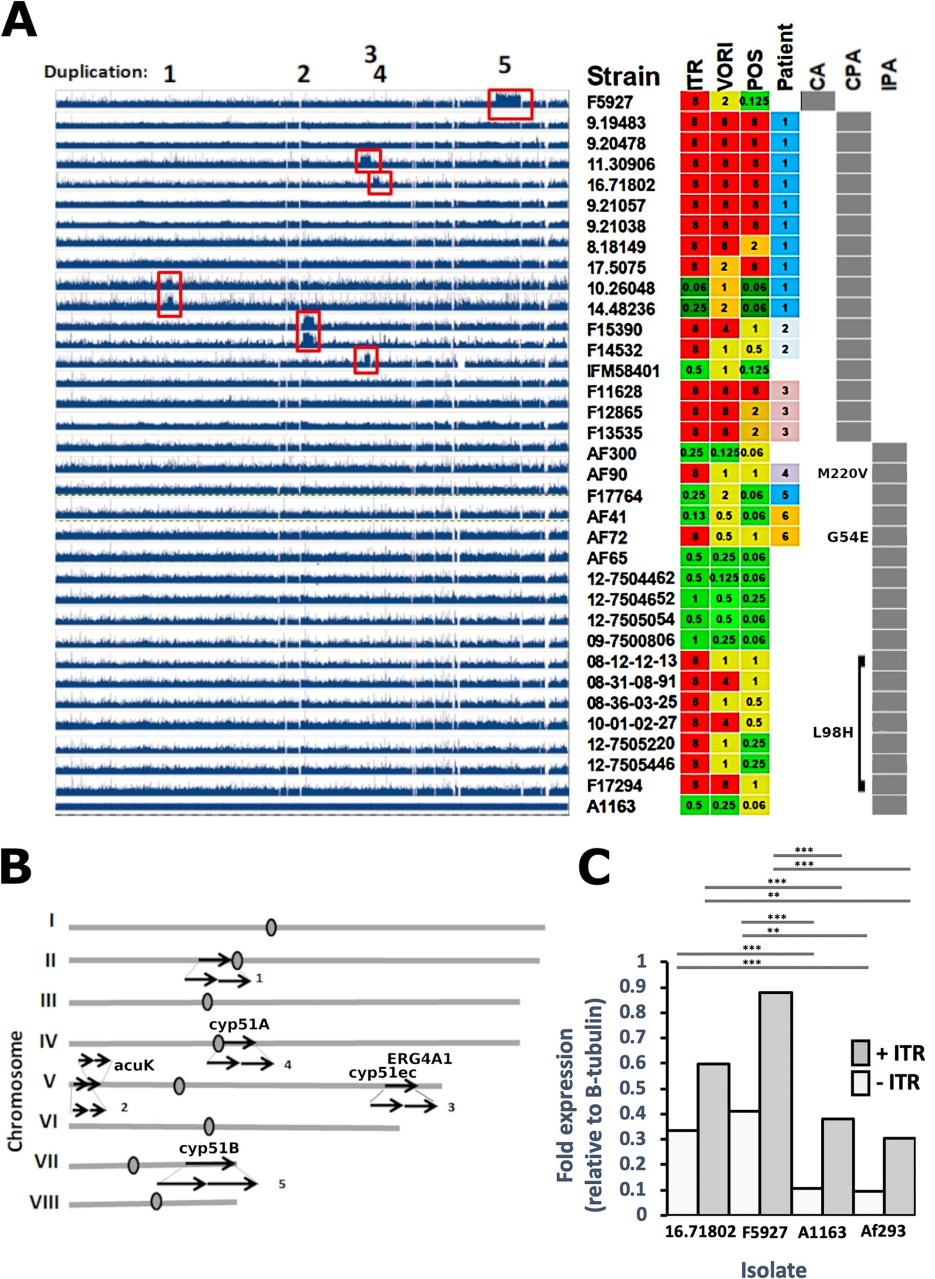
Chromosomal segment duplications in A. fumigatus CPA isolates. (A) Copy number variation plotted across the Af293 genome. Regions with 2-fold higher copy number are indicated with red boxes. Isolate names are listed next to MICs (mg/L) for itraconazole (ITR), voriconazole (VORI), and posaconazole (POS). The patient from which the isolate derives is listed in the next column and the disease. CPA, chronic pulmonary aspergillosis; CA, cerebral aspergillosis; IPA, invasive pulmonary aspergillosis. Mutations in cyp51A are indicated in white text within the gray disease marker box. (B) Chromosomal locations of duplicated regions mapped to the Af293 genome. Genes of interest are marked for each region. (C) Expression level of cyp51A in isolates with duplicated regions containing cyp51A. Experiments included three technical replicates of three biological replicates. *** indicates *P* value <0.001; ** indicates *P* value <0.01.

Genome sequences from 16 CPA, 11 environmental, and 38 A. fumigatus isolates from patients with invasive pulmonary aspergillosis (IPA) were assessed for aneuploidy. All 16 CPA isolates were obtained from six patients over the course of therapy and seven of them (43.75%) displayed aneuploidy (Fig. 1A). Of these, two identical duplications were observed in isolates F15390 and F14352 and another two identical duplications were observed in strains 10.26048 and 14.48326. Each pair of these isolates was obtained from an individual patient. No invasive or environmental isolate showed aneuploidy. Many of these duplications occurred in azole resistant CPA isolates and may account for azole resistance as duplicated chromosomal regions contain genes associated with azole tolerance or resistance such as cyp51A, cyp51B, the cyp51 electron carrier protein cyp51ec, several major facilitator superfamily (MFS), or ABC transporters and transcription factors.

In order to determine whether duplication of chromosomal segments containing *cyp*51A results in overexpression, we used RT-PCR to test gene expression of isolates with aneuploid regions containing *cyp*51A (10) and control isolates in the presence and absence of sub-MIC levels of itraconazole (Fig. 1C). We found isolates carrying duplications in *cyp*51A overexpressed these genes either in the presence of absence of azole. We have previously reported that a 2-fold overexpression of *cyp*51A is sufficient for high-level azole resistance and we note that overexpression of *cyp*51B is associated with resistance in clinical isolates although no causal relationship has yet been established (11, 12).

Resistance in IPA is often associated with *cyp*51A mutations previously associated with azole resistance (5). A single cerebral aspergillosis isolate (labeled “CA” in Fig. 1) included in the study displayed both resistance to itraconazole and aneuploidy. Chromosomal regions mapped to the Af293 genome are indicated in Fig. 1B. The list of genes contained within these regions is given in Fig. S2. Genes of potential interest are indicated in Fig. 1B.

Our study demonstrated that aneuploidy is observed in a significant proportion of azole resistant CPA isolates that do not carry a mutation in *cyp*51A (43.75%) with duplicated chromosomal regions frequently containing genes known to be involved in azole resistance. This result must be treated with caution as only one azole sensitive CPA isolate is included in the study and the isolates were taken from only six CPA patients with potential confounding effects due to lineage. We also note that *cyp*51A-mediated resistance has been previously observed in CPA, although such isolates were not deliberately excluded from this study. We therefore expect this figure might be an overestimate. Because aneuploidy in this study was confined to CPA isolates, this suggests that either particular stress environment or prolonged azole treatment is important in this form of resistance in Aspergillus. Given the small size of this study, it is likely that aneuploidy will also be observed in invasive or environmental isolates that have been exposed to stress over long periods; however, it is possible that the long-term nature of CPA may predispose to aneuploid modes of stress adaptation. Aneuploidy is well studied in model fungal systems and has been shown to arise during a wide range of conditions such as osmotic or oxidative stress. Azole resistance arising from aneuploidy has been observed in laboratory isolates of *Candida* and Cryptococcus (9, 13); however, this is the first observation of this phenomenon in Aspergillus to our knowledge.

Aspergillus fumigatus genome sequence was obtained from NCBI SRA archive for previously characterized invasive and environmental isolates (listed in [14] and Table S1). A further set of CPA isolates, was obtained from the Mycology Reference Centre Manchester and sequenced in this study, including F5927, 9.19483, 9.20478, 11.30906, 16.71802, 9.21057, 9.21038, 8.18149, 17.5075, 10.26048, 14.48236, and AF300. For these isolates, genomic DNA was extracted as described in (15). Quantity and size of DNA was assessed using gel electrophoresis to ensure quantity and minimum 50 Kb fragment size. Genomes were sequenced using an Illumina MiSEQ to a depth of >70× for all isolates in the study. MICs were determined according to the European Committee on Antimicrobial Susceptibility Testing (EUCAST) standard methodology (16).

Genome sequences were subjected to QC using Trimmomatic v0.36 (14) and CNV was assessed in both gene dependent and gene independent manner using bespoke scripts based around Bowtie 2 (17). Alignment to cDNA and overlapping 500 bp genome segments was performed as described in the Bowtie2 manual and previously exemplified in fungi (18). CNV was mapped to both A1163 and Af293 genomes and potential boundary regions were remapped to confirm duplication boundaries. Results are shown for Af293 but are the same for A1163.

Differences in gene expression of A. fumigatus cyp*51A* in the presence or absence of itraconazole was determined for strains displaying aberrant *cyp51* copy numbers compared to controls. RT–PCR for *cyp51A* was performed as previously described (10). Negative controls consisted of no-RNA or no-template wells with or without reverse transcriptase and reactions were performed in triplicate. Primers used are *cyp51A*qPCR-F (TGCAGAGAAAAGTATGGCGA) and *cyp51A* qPCR-R (CGCATTGACATCCTTGAGC). Changes in gene expression were determined using the 2^−ΔΔCt^ method. Statistical analysis using nonparametric analogous Wilcoxon tests on paired samples was performed using GraphPad Prism v9 (La Jolla, CA, USA).
