# Supplementary material for: Aneuploidy Is Associated with Azole Resistance in Aspergillus fumigatus
Source: Antimicrob Agents Chemother. 2023 Mar 28;67(4):e01253-22. doi: 10.1128/aac.01253-22 (PMC10112202; doi:10.1128/aac.01253-22)
Supplement: Supplemental file 1 — Supplemental material. Download aac.01253-22-s0001.pdf, PDF file, 0.3 MB [file aac.01253-22-s0001.pdf]

Supplementary Table 1

Sources of additional previously published isolate sequences used in this study. Clinical isolates are mostly invasive however some were cultured from additional sources included wounds, tissues (e.g., eyes, ears, nails, and brain), body fluids, spinal specimens, and unknown (10,19,20)

Isolate	Accession	Source	Azole resistance
10/01/2002	ERR769511	Clinical	Resistant
08/12/2012	ERR769506	Clinical	Resistant
08-19-02	ERR769513	Environmental	Resistant
08-19-02	ERR769510	Environmental	Susceptible
08-31-08	ERR769508	Clinical	Resistant
08-36-03	ERR769507	Clinical	Resistant
09-750080	ERR769502	Clinical	Susceptible
12-750446	ERR769504	Clinical	Susceptible
12-750465	ERR769503	Clinical	Susceptible
12-750505	ERR769505	Clinical	Susceptible
12-750522	ERR769501	Clinical	Resistant
12-750544	ERR769500	Clinical	Resistant
AFIS1878	SAMN14598471	Clinical	Resistant

AFIS2001	SAMN14598491	Clinical	Resistant
AFIS2889	SAMN14598560	Clinical	Resistant
B11930	SAMN14598570	Environmental	Resistant
B11943	SAMN14598571	Environmental	Resistant
B11951	SAMN14598572	Environmental	Resistant
AB11957	SAMN14598573	Environmental	Resistant
B11969	SAMN14598576	Environmental	Resistant
B11982	SAMN14598580	Environmental	Resistant
AF41	SRR617720	Clinical	Sensitive
AF72	SRR617721	Clinical	Sensitive
AF90	SRR617722		Resistant
AFIS2254	SAMN15356935	Clinical	Resistant
Afu1042-09	ERR769515	Clinical	Resistant
Afu166-E11	ERR769519	Environmental	Resistant,
Afu218-E11	ERR769521	Environmental	Resistant
Afu257-E11	ERR769520	Environmental	Resistant
Afu591-12	ERR769517	Clinical	Resistant
Afu942-09	ERR769514	Clinical	Resistant
AFIS1417	SAMN14598404	Clinical	Resistant
AFIS1484	SAMN14598411	Clinical	Resistant

AFIS1554	SAMN14598427	Clinical	Resistant
AFIS1874	SAMN14598469	Clinical	Resistant
AFIS1926	SAMN14598480	Clinical	Resistant
AFIS2064	SAMN14598499	Clinical	Resistant
AFIS2068	SAMN14598503	Clinical	Resistant
AFIS2105	SAMN14598509	Clinical	Resistant
AFIS2211	SAMN14598525	Clinical	Resistant
AFIS2241	SAMN14598529	Clinical	Resistant
AFIS2242	SAMN14598530	Clinical	Resistant
AFIS2288	SAMN14598535	Clinical	Resistant
AFIS2305	SAMN14598538	Clinical	Resistant
AFIS2400	SAMN14598543	Clinical	Resistant
AFIS3129	SAMN14598563	Clinical	Resistant
BB11928	SAMN14598569	Environmental	Resistant
BB11971	SAMN14598577	Environmental	Resistant
BB11973	SAMN14598578	Environmental	Resistant
F11628	SRR343149	Clinical	Resistant
F12865	SRR343150	Clinical	Resistant
F13535	SRR617726	Clinical	Resistant
F14532	SRR617731	Clinical	Resistant

F15390	SRR617733	Clinical	Resistant
F17764	SRR617745	Clinical	Resistant

Supplementary Table 2

Genes in duplicated regions marked in Figure 1

Gene ID	Genomic Location (Gene)	Product Description
Afu2g05230	Chr2_A_fumigatus_Af293:1,452,392..1,453,989(+)	Ortholog of A. nidulans FGSC A4 : AN2111, A. fumigatus Af293 : Afu6g03190, A. niger CBS 513.88 : An11g05190, A. oryzae RIB40 : AO090010000628, AO090003000332 and Aspergillus wentii : Aspwe1_0055162
Afu2g05240	Chr2_A_fumigatus_Af293:1,456,446..1,457,711(+)	Ortholog(s) have extracellular region localization
Afu2g05250	Chr2_A_fumigatus_Af293:1,458,288..1,461,192(-)	putative transcription factor
Afu2g05260	Chr2_A_fumigatus_Af293:1,464,656..1,466,072(+)	Has domain(s) with predicted FAD binding, oxidoreductase activity and role in metabolic process
Afu2g05270	Chr2_A_fumigatus_Af293:1,467,280..1,468,332(-)	Ortholog of Aspergillus fumigatus A1163 : AFUB_022300
Afu2g05280	Chr2_A_fumigatus_Af293:1,468,485..1,469,128(-)	protein of unknown function
Afu2g05290	Chr2_A_fumigatus_Af293:1,470,208..1,472,390(-)	Ortholog of A. nidulans FGSC A4 : AN3976, A. niger CBS 513.88 : An02g11140, Neosartorya fischeri NRRL 181 : NFIA_082050, Aspergillus wentii : Aspwe1_0592927 and Aspergillus versicolor : Aspve1_0080485
Afu2g05300	Chr2_A_fumigatus_Af293:1,472,472..1,473,777(-)	Ortholog of Aspergillus fumigatus A1163 : AFUB_022330
Afu2g05310	Chr2_A_fumigatus_Af293:1,474,504..1,479,018(-)	Has domain(s) with predicted DNA binding, RNA polymerase II transcription factor activity, sequence-specific DNA binding, zinc ion binding activity and role in regulation of transcription, DNA-templated, transcription, DNA-templated
Afu2g05325	Chr2_A_fumigatus_Af293:1,480,858..1,484,987(-)	Has domain(s) with predicted role in transmembrane transport and integral component of membrane localization
Afu2g05340	Chr2_A_fumigatus_Af293:1,486,634..1,489,175(+)	Essential 1,3-beta-glucanosyltransferase, GPI-anchored to the plasma membrane
Afu2g05350	Chr2_A_fumigatus_Af293:1,489,567..1,491,076(+)	Putative MFS transporter
Afu2g05360	Chr2_A_fumigatus_Af293:1,491,344..1,493,915(-)	Putative C6 transcription factor
Afu2g05370	Chr2_A_fumigatus_Af293:1,495,691..1,496,482(+)	Ortholog of Aspergillus fumigatus A1163 : AFUB_022400
Afu2g05380	Chr2_A_fumigatus_Af293:1,498,429..1,500,925(+)	Has domain(s) with predicted DNA binding, RNA polymerase II transcription factor activity, sequence-specific DNA binding, zinc ion binding activity and role in regulation of transcription, DNA-templated, transcription, DNA-templated
Afu2g05390	Chr2_A_fumigatus_Af293:1,504,321..1,509,831(+)	Has domain(s) with predicted role in intracellular signal transduction
Afu2g05400	Chr2_A_fumigatus_Af293:1,510,443..1,512,936(+)	Ortholog(s) have hydrolase activity, hydrolyzing O-glycosyl compounds activity and role in xyloglucan metabolic process
Afu2g05410	Chr2_A_fumigatus_Af293:1,513,346..1,516,972(-)	Ortholog of A. nidulans FGSC A4 : AN7504, A. oryzae RIB40 : AO090001000651, Aspergillus wentii : Aspwe1_0121527, Aspergillus sydowii : Aspsy1_0152683 and Aspergillus terreus NIH2624 : ATET_06731
Afu2g05420	Chr2_A_fumigatus_Af293:1,518,008..1,520,305(-)	Ortholog(s) have nucleolus localization
Afu2g05430	Chr2_A_fumigatus_Af293:1,520,509..1,522,677(-)	Ortholog(s) have uridine kinase activity, role in pyrimidine-containing compound salvage and cell division site, cytosol, mitotic spindle pole body, nucleus localization
Afu2g05440	Chr2_A_fumigatus_Af293:1,522,761..1,524,497(+)	Ortholog(s) have cytosol, nucleus localization
Afu2g05450	Chr2_A_fumigatus_Af293:1,524,596..1,527,501(-)	Mitochondrial NADH dehydrogenase involved in oxidative phosphorylation
Afu2g05470	Chr2_A_fumigatus_Af293:1,527,534..1,533,669(-)	Ortholog(s) have role in attachment of mitotic spindle microtubules to kinetochore, cohesin localization to chromatin, mitotic sister chromatid cohesion and negative regulation of G0 to G1 transition, more
Afu2g05480	Chr2_A_fumigatus_Af293:1,533,897..1,534,392(+)	Ortholog(s) have RNA polymerase I activity and role in termination of RNA polymerase I transcription, transcription of nuclear large rRNA transcript from RNA polymerase I promoter
Afu2g05490	Chr2_A_fumigatus_Af293:1,534,498..1,535,686(-)	Ortholog(s) have deoxyhypusine monooxygenase activity, role in microtubule cytoskeleton organization, mitochondrion distribution, peptidyl-lysine modification to peptidyl-hypusine and cytosol, nucleus localization
Afu2g05500	Chr2_A_fumigatus_Af293:1,535,737..1,537,421(-)	Ortholog(s) have oxidoreductase activity and mitochondrion localization
Afu2g05510	Chr2_A_fumigatus_Af293:1,537,429..1,538,546(+)	Ortholog(s) have proton-transporting ATP synthase activity, rotational mechanism activity and role in ATP synthesis coupled proton transport
Afu2g05520	Chr2_A_fumigatus_Af293:1,538,674..1,543,847(+)	Ortholog of A. nidulans FGSC A4 : AN7496, A. niger CBS 513.88 : An02g13860, A. oryzae RIB40 : AO090001000663, Aspergillus wentii : Aspwe1_0121457 and Aspergillus sydowii : Aspsy1_0058467
Afu2g05530	Chr2_A_fumigatus_Af293:1,543,958..1,546,325(-)	Ortholog(s) have H4 histone acetyltransferase activity, histone acetyltransferase activity (H3-K4 specific), peptide N-acetyltransferase activity
Afu2g05540	Chr2_A_fumigatus_Af293:1,546,346..1,549,205(-)	Ortholog(s) have DNA binding activity, role in mRNA cis splicing, via spliceosome and Prp19 complex, cytosol, spliceosomal complex localization
Afu2g05550	Chr2_A_fumigatus_Af293:1,549,332..1,550,996(+)	Ortholog of A. nidulans FGSC A4 : AN10943, A. niger CBS 513.88 : An02g13890, A. oryzae RIB40 : AO090001000666, Neosartorya fischeri NRRL 181 : NFIA_082270 and Aspergillus clavatus NRRL 1 : ACLA_089150
Afu2g05560	Chr2_A_fumigatus_Af293:1,551,103..1,552,239(+)	Putative exonuclease
Afu2g05570	Chr2_A_fumigatus_Af293:1,552,642..1,553,462(+)	Ortholog of Aspergillus fumigatus A1163 : AFUB_022590
Afu2g05580	Chr2_A_fumigatus_Af293:1,553,837..1,555,318(+)	Ortholog(s) have RNA 7-methylguanosine cap binding, exoribonuclease activator activity, m7G(5')pppN diphosphatase activity
Afu2g05590	Chr2_A_fumigatus_Af293:1,555,349..1,556,329(+)	Ortholog(s) have ubiquitin-specific protease activity, role in protein deubiquitination and cytosol, nucleus localization
Afu2g05600	Chr2_A_fumigatus_Af293:1,556,615..1,557,982(-)	Ortholog(s) have cytosol localization
Afu2g05610	Chr2_A_fumigatus_Af293:1,558,179..1,563,688(-)	Has domain(s) with predicted RNA binding, nucleic acid binding, nucleotide binding activity
Afu2g05620	Chr2_A_fumigatus_Af293:1,565,153..1,567,800(+)	Ortholog(s) have histone binding activity, role in histone exchange and Swr1 complex, cytoplasm localization
Afu2g05630	Chr2_A_fumigatus_Af293:1,569,298..1,573,439(+)	Ortholog of A. nidulans FGSC A4 : AN7476, A. niger CBS 513.88 : An02g14010, A. oryzae RIB40 : AO090001000675, Aspergillus wentii : Aspwe1_0120551 and Aspergillus sydowii : Aspsy1_0058449
Afu2g05635	Chr2_A_fumigatus_Af293:1,573,966..1,574,332(-)	Ortholog of Neosartorya fischeri NRRL 181 : NFIA_082360 and Aspergillus fumigatus A1163 : AFUB_022660
Afu2g05640	Chr2_A_fumigatus_Af293:1,577,611..1,580,102(+)	Ortholog of A. niger CBS 513.88 : An02g14070, Neosartorya fischeri NRRL 181 : NFIA_082370, Aspergillus niger ATCC 1015 : 52472-mRNA and Aspergillus zonatus : Aspzo1_1187186
Afu2g05650	Chr2_A_fumigatus_Af293:1,580,139..1,582,404(-)	Ortholog(s) have asparagine-tRNA ligase activity, role in asparaginyl-tRNA aminoacylation and cytosol localization
Afu2g05660	Chr2_A_fumigatus_Af293:1,583,515..1,583,824(+)	Ortholog of Aspergillus fumigatus A1163 : AFUB_022690
Afu2g05670	Chr2_A_fumigatus_Af293:1,584,647..1,587,163(+)	Ortholog(s) have poly(U) RNA binding activity, role in negative regulation of induction of conjugation with cellular fusion by negative regulation of transcription from RNA polymerase II promoter and cytosol localization
Afu2g05680	Chr2_A_fumigatus_Af293:1,588,053..1,589,891(+)	Ortholog of A. nidulans FGSC A4 : AN7481, A. niger CBS 513.88 : An02g14110, A. oryzae RIB40 : AO090001000682, Aspergillus wentii : Aspwe1_0055195 and Aspergillus sydowii : Aspsy1_0153107
Afu2g05690	Chr2_A_fumigatus_Af293:1,591,215..1,591,904(-)	Ortholog of A. nidulans AN7482
Afu2g05700	Chr2_A_fumigatus_Af293:1,605,250..1,607,039(-)	Ortholog of A. oryzae RIB40 : AO090001000687, Neosartorya fischeri NRRL 181 : NFIA_082420, Aspergillus clavatus NRRL 1 : ACLA_068640 and Aspergillus sydowii : Aspsy1_0090059
Afu2g05710	Chr2_A_fumigatus_Af293:1,607,602..1,609,176(-)	Ortholog of A. nidulans FGSC A4 : AN4092, AN7483, A. oryzae RIB40 : AO090001000688, Neosartorya fischeri NRRL 181 : NFIA_082430 and Aspergillus versicolor : Aspve1_0043503
Afu2g05720	Chr2_A_fumigatus_Af293:1,609,920..1,610,516(+)	Ortholog of A. nidulans FGSC A4 : AN7484, A. niger CBS 513.88 : An02g14170, A. oryzae RIB40 : AO090001000689, Aspergillus wentii : Aspwe1_0046318 and Aspergillus sydowii : Aspsy1_0153479
Afu2g05730	Chr2_A_fumigatus_Af293:1,611,708..1,615,086(+)	Putative siderophore transporter
Afu2g05740	Chr2_A_fumigatus_Af293:1,616,839..1,619,693(+)	Putative Rho-type GTPase
Afu2g05750	Chr2_A_fumigatus_Af293:1,619,718..1,621,557(-)	Ortholog(s) have endoplasmic reticulum, fungal-type vacuole localization
Afu2g05760	Chr2_A_fumigatus_Af293:1,621,987..1,623,626(+)	Putative beta-ketoacyl synthase
Afu2g05770	Chr2_A_fumigatus_Af293:1,624,008..1,625,427(+)	Ortholog(s) have SAGA complex localization
Afu2g05780	Chr2_A_fumigatus_Af293:1,626,128..1,627,375(+)	Ortholog(s) have mRNA guanylyltransferase activity, role in 7-methylguanosine RNA capping and nucleus localization
Afu2g05790	Chr2_A_fumigatus_Af293:1,627,793..1,629,694(-)	Oligosaccharyl transferase alpha subunit
Afu2g05800	Chr2_A_fumigatus_Af293:1,629,700..1,632,631(+)	Ortholog(s) have cytoplasm localization

Afu2g05810	Chr2_A_fumigatus_Af293:1,632,693..1,634,195(-)	Putative dienelactone hydrolase
Afu2g05820	Chr2_A_fumigatus_Af293:1,634,833..1,635,629(-)	Ortholog(s) have riboflavin kinase activity, zinc ion binding activity, role in FMN biosynthetic process, riboflavin metabolic process and cytosol, mitochondrial inner membrane, nucleus localization
Afu2g05830	Chr2_A_fumigatus_Af293:1,636,448..1,638,778(-)	Putative transcription factor involved in regulation of gluconeogenesis and acquisition of iron
Afu2g05840	Chr2_A_fumigatus_Af293:1,641,567..1,643,827(-)	Putative MFS multidrug transporter
Afu2g05850	Chr2_A_fumigatus_Af293:1,644,731..1,647,324(-)	Ortholog(s) have role in double-strand break repair via homologous recombination, mitotic sister chromatid cohesion and horsetail nucleus leading edge, nuclear mitotic cohesin complex localization
Afu2g05860	Chr2_A_fumigatus_Af293:1,647,574..1,651,778(+)	Putative golgi apparatus Ca2+/Mn2+ P-type ATPase
Afu2g05870	Chr2_A_fumigatus_Af293:1,653,255..1,653,580(+)	Ortholog of Aspergillus fumigatus A1163 : AFUB_022910
Afu2g05880	Chr2_A_fumigatus_Af293:1,654,974..1,656,856(+)	Putative ammonium transporter
Afu2g05890	Chr2_A_fumigatus_Af293:1,657,282..1,660,256(-)	Ortholog(s) have role in CVT pathway, establishment of cell polarity, filamentous growth of a population of unicellular organisms, pexophagy, retrograde transport, vesicle recycling within Golgi
Afu2g05900	Chr2_A_fumigatus_Af293:1,660,444..1,662,493(+)	Ortholog(s) have protein deacetylase activity and role in chromatin silencing at centromere outer repeat region, chromatin silencing at rDNA, chromatin silencing at telomere, histone H3-K9 deacetylation, phenotypic switching
Afu2g05910	Chr2_A_fumigatus_Af293:1,662,852..1,664,600(+)	Putative hexokinase
Afu2g05920	Chr2_A_fumigatus_Af293:1,665,690..1,666,031(+)	Ortholog of Neosartorya fischeri NRRL 181 : NFIA_082640 and Aspergillus fumigatus A1163 : AFUB_022960
Afu2g05930	Chr2_A_fumigatus_Af293:1,666,230..1,668,765(-)	Ortholog(s) have small-subunit processome localization
Afu2g05940	Chr2_A_fumigatus_Af293:1,668,966..1,670,774(-)	Ortholog(s) have fungal-type vacuole membrane localization
Afu2g05950	Chr2_A_fumigatus_Af293:1,670,845..1,671,953(-)	Ortholog(s) have U3 snoRNA binding, U4 snRNA binding activity and role in mRNA splicing, via spliceosome, maturation of SSU-rRNA from tricistronic rRNA transcript (SSU-rRNA, 5.8S rRNA, LSU-rRNA)
Afu2g05960	Chr2_A_fumigatus_Af293:1,672,720..1,673,466(-)	Has domain(s) with predicted nucleic acid binding, nucleotide binding activity
Afu2g05970	Chr2_A_fumigatus_Af293:1,674,090..1,680,055(+)	Has domain(s) with predicted zinc ion binding activity
Afu2g05980	Chr2_A_fumigatus_Af293:1,680,066..1,683,275(-)	Ortholog of S. cerevisiae : TDA6, A. nidulans FGSC A4 : AN7454, A. niger CBS 513.88 : An02g14280, A. oryzae RIB40 : AO090001000716 and Aspergillus wentii : Aspwe1_0055229
Afu2g05990	Chr2_A_fumigatus_Af293:1,683,648..1,690,695(+)	Ortholog of A. nidulans FGSC A4 : AN7453, A. niger CBS 513.88 : An02g14270, Aspergillus wentii : Aspwe1_0032863, Aspergillus sydowii : Aspsy1_0090031 and Aspergillus terreus NIH2624 : ATET_06789
Afu2g06000	Chr2_A_fumigatus_Af293:1,696,007..1,700,187(+)	Putative NAD+ dependent glutamate dehydrogenase
Afu2g06010	Chr2_A_fumigatus_Af293:1,701,552..1,702,608(+)	Ortholog of Aspergillus fumigatus A1163 : AFUB_023090
Afu2g06020	Chr2_A_fumigatus_Af293:1,702,686..1,705,138(-)	Ortholog(s) have role in mitochondrial respiratory chain complex I assembly
Afu2g06030	Chr2_A_fumigatus_Af293:1,705,489..1,706,456(+)	Ortholog(s) have structural constituent of ribosome activity and mitochondrial small ribosomal subunit localization
Afu2g06040	Chr2_A_fumigatus_Af293:1,706,470..1,708,794(+)	Putative actin cortical patch component
Afu2g06050	Chr2_A_fumigatus_Af293:1,709,213..1,712,606(+)	Ortholog of A. nidulans FGSC A4 : AN10954, A. niger CBS 513.88 : An02g14630, A. oryzae RIB40 : AO090001000720, AO090113000187 and Aspergillus tubingensis : Asptu1_0042313
Afu2g06060	Chr2_A_fumigatus_Af293:1,713,004..1,714,478(+)	Ortholog(s) have transcription coactivator activity, role in histone acetylation, transcription from RNA polymerase II promoter and Ada2/Gcn5/Ada3 transcription activator complex, SAGA complex, SLIK (SAGA-like) complex localization
Afu2g06070	Chr2_A_fumigatus_Af293:1,715,248..1,718,524(-)	Ortholog(s) have role in mRNA cis splicing, via spliceosome and U4/U6 x U5 tri-snRNP complex localization
Afu2g06080	Chr2_A_fumigatus_Af293:1,718,679..1,722,146(-)	Ortholog(s) have cytosol, nucleus localization
Afu2g06090	Chr2_A_fumigatus_Af293:1,722,228..1,725,911(+)	Ortholog(s) have nucleus localization
Afu2g06100	Chr2_A_fumigatus_Af293:1,726,894..1,729,986(-)	Ortholog of A. nidulans FGSC A4 : AN7443, A. niger CBS 513.88 : An02g14710, A. oryzae RIB40 : AO090001000726, Aspergillus wentii : Aspwe1_0110234 and Aspergillus sydowii : Aspsy1_0152786
Afu2g06110	Chr2_A_fumigatus_Af293:1,733,688..1,735,660(+)	Ortholog(s) have role in chromatin silencing at centromere, histone acetylation, histone exchange, mitotic sister chromatid biorientation and Ino80 complex, NuA4 histone acetyltransferase complex, Swr1 complex localization
Afu2g06120	Chr2_A_fumigatus_Af293:1,735,733..1,738,887(-)	Ortholog of A. nidulans FGSC A4 : AN7433, A. niger CBS 513.88 : An02g14760, Aspergillus wentii : Aspwe1_0110312, Aspergillus sydowii : Aspsy1_0153514 and Aspergillus terreus NIH2624 : ATET_05606
Afu2g06130	Chr2_A_fumigatus_Af293:1,740,097..1,742,242(-)	Has domain(s) with predicted DNA binding, zinc ion binding activity, role in transcription, DNA-templated and nucleus localization
Afu2g06140	Chr2_A_fumigatus_Af293:1,742,740..1,744,237(+)	uracil DNA N-glycosylase, putative
Afu2g06150	Chr2_A_fumigatus_Af293:1,744,786..1,746,985(-)	Putative protein disulfide isomerase
Afu2g06160	Chr2_A_fumigatus_Af293:1,747,870..1,750,485(-)	Ortholog of Aspergillus fumigatus A1163 : AFUB_023240
Afu2g06170	Chr2_A_fumigatus_Af293:1,752,414..1,755,226(-)	Ortholog(s) have L-amino acid transmembrane transporter activity, role in L-alpha-amino acid transmembrane transport and fungal-type vacuole membrane localization
Afu2g06180	Chr2_A_fumigatus_Af293:1,755,388..1,759,465(+)	Has domain(s) with predicted RNA polymerase II transcription cofactor activity, role in regulation of transcription from RNA polymerase II promoter and mediator complex localization
Afu2g06190	Chr2_A_fumigatus_Af293:1,759,842..1,762,130(-)	Ortholog(s) have DNA-directed DNA polymerase activity, double-stranded DNA binding, single-stranded DNA binding activity
Afu2g06200	Chr2_A_fumigatus_Af293:1,762,681..1,764,118(+)	Ortholog(s) have role in CENP-A containing nucleosome assembly, chromatin maintenance, chromatin silencing at centromere, mitotic sister chromatid segregation and nucleoplasm localization
Afu2g06205	Chr2_A_fumigatus_Af293:1,764,499..1,765,197(-)	Ortholog of A. niger CBS 513.88 : An02g14850, A. oryzae RIB40 : AO090001000738, Aspergillus wentii : Aspwe1_0028348, Aspergillus clavatus NRRL 1 : ACLA_068110 and Aspergillus niger ATCC 1015 : 175113-mRNA
Afu2g06220	Chr2_A_fumigatus_Af293:1,791,120..1,794,643(-)	Ortholog(s) have nucleus localization
Afu2g06230	Chr2_A_fumigatus_Af293:1,794,669..1,796,803(-)	Ortholog(s) have glutaminase activity, imidazoleglycerol-phosphate synthase activity, role in histidine biosynthetic process and cytosol, nucleus localization
Afu2g06240	Chr2_A_fumigatus_Af293:1,797,448..1,799,650(-)	Ortholog(s) have dCMP deaminase activity, role in dTMP biosynthetic process, dUMP biosynthetic process and cytosol, nucleus localization
Afu2g06250	Chr2_A_fumigatus_Af293:1,799,951..1,802,480(+)	Ortholog(s) have Atg12 activating enzyme activity, Atg8 activating enzyme activity
Afu2g06260	Chr2_A_fumigatus_Af293:1,802,669..1,805,601(+)	Ortholog of Aspergillus fumigatus A1163 : AFUB_023340

Gene ID	Genomic Location (Gene)	Product Description
Afu5g00100	Chr5_A_fumigatus_Af293:1,474..2,924(-)	Ortholog of A. nidulans FGSC A4 : AN9205, AN9209, A. fumigatus Af293 : Afu8g01840, Afu7g06523, A. niger CBS 513.88 : An05g01030, An13g03050, An16g01380 and A. oryzae RIB40 : AO090012000290, AO090011000069
Afu5g00110	Chr5_A_fumigatus_Af293:3,119..5,290(-)	Putative squalene-hopene-cyclase
Afu5g00120	Chr5_A_fumigatus_Af293:5,839..7,498(+)	Putative cytochrome P450 oxidoreductase/alkane hydroxylase
Afu5g00130	Chr5_A_fumigatus_Af293:8,045..9,220(+)	Ortholog of A. nidulans FGSC A4 : AN5461, A. niger CBS 513.88 : An01g00570, Aspergillus wentii : Aspwe1_0022994, Aspergillus versicolor : Aspve1_0039792 and Aspergillus clavatus NRRL 1 : ACLA_024300
Afu5g00135	Chr5_A_fumigatus_Af293:9,314..10,498(-)	Ortholog(s) have transcription factor activity, RNA polymerase II core promoter proximal region sequence-specific binding activity
Afu5g00145	Chr5_A_fumigatus_Af293:15,749..16,327(-)	Conserved protein of unknown function
Afu5g00150	Chr5_A_fumigatus_Af293:17,759..18,612(+)	Has domain(s) with predicted hydrolase activity, acting on ester bonds activity and role in biosynthetic process
Afu5g00155	Chr5_A_fumigatus_Af293:19,084..21,497(-)	protein of unknown function
Afu5g00160	Chr5_A_fumigatus_Af293:24,489..25,947(+)	Has domain(s) with predicted role in transmembrane transport and integral component of membrane localization
Afu5g00170	Chr5_A_fumigatus_Af293:32,869..33,471(-)	protein of unknown function
Afu5g00180	Chr5_A_fumigatus_Af293:34,623..35,952(+)	Has domain(s) with predicted membrane localization
Afu5g00190	Chr5_A_fumigatus_Af293:37,838..38,262(-)	Ortholog of Aspergillus acidus : Aspfo1_0144348
Afu5g00200	Chr5_A_fumigatus_Af293:41,963..42,498(-)	protein of unknown function
Afu5g00210	Chr5_A_fumigatus_Af293:46,426..46,989(-)	protein of unknown function
Afu5g00220	Chr5_A_fumigatus_Af293:49,116..49,679(+)	Ortholog of Aspergillus kawachii : Aspka1_0183260 and Aspergillus niger ATCC 1015 : 184254-mRNA
Afu5g00230	Chr5_A_fumigatus_Af293:50,766..52,564(+)	protein of unknown function
Afu5g00240	Chr5_A_fumigatus_Af293:59,436..59,855(-)	protein of unknown function
Afu5g00250	Chr5_A_fumigatus_Af293:60,204..61,033(-)	protein of unknown function
Afu5g00260	Chr5_A_fumigatus_Af293:63,859..64,734(+)	Has domain(s) with predicted oxidoreductase activity and role in oxidation-reduction process
Afu5g00280	Chr5_A_fumigatus_Af293:78,168..80,411(+)	Has domain(s) with predicted substrate-specific transmembrane transporter activity, transmembrane transporter activity, role in transmembrane transport and integral component of membrane, membrane localization
Afu5g00290	Chr5_A_fumigatus_Af293:81,096..84,172(-)	Has domain(s) with predicted RNA polymerase II transcription factor activity, sequence-specific DNA binding, zinc ion binding activity, role in regulation of transcription, DNA-templated and nucleus localization
Afu5g00300	Chr5_A_fumigatus_Af293:85,519..87,104(-)	Has domain(s) with predicted oxidoreductase activity, transferase activity, transferring acyl groups other than amino-acyl groups, zinc ion binding activity and role in oxidation-reduction process
Afu5g00310	Chr5_A_fumigatus_Af293:87,674..90,270(-)	Putative flavin-containing monooxygenase
Afu5g00320	Chr5_A_fumigatus_Af293:91,159..91,528(-)	Ortholog of Aspergillus fumigatus A1163 : AFUB_048770
Afu5g00330	Chr5_A_fumigatus_Af293:91,964..93,063(+)	Has domain(s) with predicted ATP binding, catalytic activity
Afu5g00340	Chr5_A_fumigatus_Af293:93,541..98,243(-)	Has domain(s) with predicted ATP binding, ATPase activity, ATPase activity, coupled to transmembrane movement of substances, nucleoside-triphosphatase activity, nucleotide binding activity and role in transmembrane transport
Afu5g00352	Chr5_A_fumigatus_Af293:98,367..100,516(+)	Has domain(s) with predicted catalytic activity and role in metabolic process
Afu5g00370	Chr5_A_fumigatus_Af293:102,374..103,829(+)	Has domain(s) with predicted role in transmembrane transport and membrane localization
Afu5g00380	Chr5_A_fumigatus_Af293:105,691..107,442(-)	Ortholog(s) have intracellular localization
Afu5g00390	Chr5_A_fumigatus_Af293:109,419..112,168(+)	Ortholog of A. nidulans FGSC A4 : AN3986, AN0094, A. fumigatus Af293 : Afu5g12080, Afu7g04811 and A. niger CBS 513.88 : An01g14540, An18g02250, An13g01370, An12g06190, An02g04420
Afu5g00400	Chr5_A_fumigatus_Af293:113,522..114,899(-)	Has domain(s) with predicted catalytic activity, pyridoxal phosphate binding activity and role in biosynthetic process
Afu5g00410	Chr5_A_fumigatus_Af293:115,692..117,567(-)	Ortholog(s) have intracellular localization
Afu5g00420	Chr5_A_fumigatus_Af293:118,071..119,830(+)	Has domain(s) with predicted role in transmembrane transport and integral component of membrane localization
Afu5g00430	Chr5_A_fumigatus_Af293:121,699..123,740(-)	Has domain(s) with predicted role in transmembrane transport and integral component of membrane localization
Afu5g00435	Chr5_A_fumigatus_Af293:124,091..125,971(+)	Has domain(s) with predicted RNA polymerase II transcription factor activity, sequence-specific DNA binding, zinc ion binding activity, role in regulation of transcription, DNA-templated and nucleus localization
Afu5g00440	Chr5_A_fumigatus_Af293:126,018..126,892(-)	Has domain(s) with predicted N-acetyltransferase activity
Afu5g00460	Chr5_A_fumigatus_Af293:129,099..130,723(-)	Has domain(s) with predicted oxidoreductase activity and role in oxidation-reduction process
Afu5g00470	Chr5_A_fumigatus_Af293:132,072..133,983(+)	General amidase
Afu5g00480	Chr5_A_fumigatus_Af293:134,985..137,235(+)	Has domain(s) with predicted hydrolase activity, hydrolyzing O-glycosyl compounds activity and role in carbohydrate metabolic process
Afu5g00490	Chr5_A_fumigatus_Af293:139,561..141,100(+)	Ortholog of Aspergillus fumigatus A1163 : AFUB_048950
Afu5g00500	Chr5_A_fumigatus_Af293:144,344..146,547(-)	Has domain(s) with predicted transmembrane transporter activity, role in transmembrane transport and integral component of membrane localization
Afu5g00510	Chr5_A_fumigatus_Af293:147,684..149,094(-)	Ortholog of A. niger CBS 513.88 : An06g02400, Aspergillus brasiliensis : Aspbr1_0031922, Neosartorya fischeri NRRL 181 : NFIA_042000 and Aspergillus fumigatus A1163 : AFUB_048980
Afu5g00520	Chr5_A_fumigatus_Af293:149,332..151,518(+)	Has domain(s) with predicted DNA binding, RNA polymerase II transcription factor activity, sequence-specific DNA binding, zinc ion binding activity and role in regulation of transcription, DNA-templated, transcription, DNA-templated
Afu5g00530	Chr5_A_fumigatus_Af293:151,569..153,420(-)	Ortholog(s) have inulinase activity and role in carbohydrate metabolic process
Afu5g00540	Chr5_A_fumigatus_Af293:154,401..155,764(-)	Ortholog of A. fumigatus Af293 : Afu3g14910, Neosartorya fischeri NRRL 181 : NFIA_041970, NFIA_062340, Aspergillus wentii : Aspwe1_0435722 and Aspergillus versicolor : Aspve1_0042882
Afu5g00550	Chr5_A_fumigatus_Af293:157,167..158,726(-)	Has domain(s) with predicted catalytic activity, hydrolase activity, hydrolyzing O-glycosyl compounds activity and role in carbohydrate metabolic process
Afu5g00560	Chr5_A_fumigatus_Af293:160,463..161,658(-)	Ortholog of Neosartorya fischeri NRRL 181 : NFIA_041940 and Aspergillus fumigatus A1163 : AFUB_049030
Afu5g00570	Chr5_A_fumigatus_Af293:162,664..165,353(+)	Ortholog of Aspergillus fumigatus A1163 : AFUB_049040
Afu5g00580	Chr5_A_fumigatus_Af293:165,766..167,189(-)	Ortholog of Neosartorya fischeri NRRL 181 : NFIA_041910 and Aspergillus fumigatus A1163 : AFUB_049050
Afu5g00590	Chr5_A_fumigatus_Af293:167,534..168,580(-)	Ortholog of A. niger CBS 513.88 : An15g07140, An13g01800, Neosartorya fischeri NRRL 181 : NFIA_023890, NFIA_041900 and Aspergillus wentii : Aspwe1_0039567, Aspwe1_0040764, Aspwe1_0044902
Afu5g00600	Chr5_A_fumigatus_Af293:169,273..170,037(-)	Ortholog(s) have extracellular region localization
Afu5g00610	Chr5_A_fumigatus_Af293:170,630..172,252(-)	Has domain(s) with predicted peroxidase activity
Afu5g00620	Chr5_A_fumigatus_Af293:174,613..176,319(+)	Ortholog of Aspergillus fumigatus A1163 : AFUB_049090
Afu5g00630	Chr5_A_fumigatus_Af293:177,277..179,441(+)	Has domain(s) with predicted flavin adenine dinucleotide binding, oxidoreductase activity, acting on CH-OH group of donors activity and role in oxidation-reduction process
Afu5g00640	Chr5_A_fumigatus_Af293:179,606..180,880(-)	Ortholog(s) have glyoxysome localization
Afu5g00650	Chr5_A_fumigatus_Af293:182,686..184,205(-)	protein of unknown function
Afu5g00660	Chr5_A_fumigatus_Af293:188,199..189,638(-)	Ortholog of Aspergillus fumigatus A1163 : AFUB_049130

Gene ID	Genomic Location (Gene)	Product Description
Afu5g00800	Chr5_A_fumigatus_Af293:219,876..222,265(+)	Ortholog of Neosartorya fischeri NRRL 181 : NFIA_041390 and Aspergillus fumigatus A1163 : AFUB_049260
Afu5g00810	Chr5_A_fumigatus_Af293:223,385..224,504(+)	Ortholog of Neosartorya fischeri NRRL 181 : NFIA_041360, Aspergillus fumigatus A1163 : AFUB_049270 and Aspergillus clavatus NRRL 1 : ACLA_004420
Afu5g00820	Chr5_A_fumigatus_Af293:226,212..228,359(+)	Ortholog of A. fumigatus Af293 : Afu6g14570, A. oryzae RIB40 : AO090009000539, Aspergillus wentii : Aspwe1_0037901, Aspergillus sydowii : Aspsy1_0044497 and Aspergillus terreus NIH2624 : ATET_07141
Afu5g00830	Chr5_A_fumigatus_Af293:228,632..230,413(-)	Ortholog of A. nidulans FGSC A4 : AN10390, AN3286, A. fumigatus Af293 : Afu4g01280, A. niger CBS 513.88 : An12g00980, An11g04090, An06g00150 and A. oryzae RIB40 : AO090138000050, AO090010000179
Afu5g00840	Chr5_A_fumigatus_Af293:230,969..232,898(-)	Ortholog of A. nidulans FGSC A4 : AN5639, AN2587, AN9444, AN7395, A. niger CBS 513.88 : An03g01000, A. oryzae RIB40 : AO090102000018 and Aspergillus wentii : Aspwe1_0065350, Aspwe1_0153928, Aspwe1_0178709
Afu5g00860	Chr5_A_fumigatus_Af293:234,649..235,431(-)	protein of unknown function
Afu5g00870	Chr5_A_fumigatus_Af293:236,240..236,663(+)	Ortholog of Aspergillus fumigatus A1163 : AFUB_049320
Afu5g00890	Chr5_A_fumigatus_Af293:237,323..238,237(+)	protein of unknown function
Afu5g00900	Chr5_A_fumigatus_Af293:238,540..239,406(+)	Putative RgsA paralog
Afu5g00910	Chr5_A_fumigatus_Af293:239,708..240,114(-)	Ortholog of A. nidulans FGSC A4 : AN1567, Neosartorya fischeri NRRL 181 : NFIA_041260, Aspergillus wentii : Aspwe1_0149298 and Aspergillus versicolor : Aspve1_0045271, Aspve1_0151961
Afu5g00920	Chr5_A_fumigatus_Af293:241,535..242,761(+)	Ortholog(s) have intracellular localization
Afu5g00930	Chr5_A_fumigatus_Af293:242,866..244,662(+)	Ortholog(s) have L-proline transmembrane transporter activity, role in proline transport and fungal-type vacuole, plasma membrane localization
Afu5g00940	Chr5_A_fumigatus_Af293:244,784..245,314(-)	protein of unknown function
Afu5g00950	Chr5_A_fumigatus_Af293:245,858..247,667(+)	Has domain(s) with predicted RNA polymerase II transcription factor activity, sequence-specific DNA binding, transcription factor activity, sequence-specific DNA binding, zinc ion binding activity
Afu5g00960	Chr5_A_fumigatus_Af293:247,752..249,860(-)	Ortholog of A. nidulans FGSC A4 : AN4040, AN7213, A. niger CBS 513.88 : An18g01180, An10g00910, An11g01220, An01g11560 and A. oryzae RIB40 : AO090026000375, AO090023000615, AO090166000079
Afu5g00975	Chr5_A_fumigatus_Af293:249,921..250,620(+)	Ortholog(s) have extracellular region localization
Afu5g00980	Chr5_A_fumigatus_Af293:254,324..255,862(+)	Has domain(s) with predicted role in transmembrane transport and integral component of membrane localization
Afu5g00990	Chr5_A_fumigatus_Af293:256,002..256,245(+)	Ortholog of Aspergillus fumigatus A1163 : AFUB_049430
Afu5g01000	Chr5_A_fumigatus_Af293:256,276..256,850(+)	Putative oxidoreductase, 2OG-Fe(II) oxygenase family protein
Afu5g01005	Chr5_A_fumigatus_Af293:257,649..265,835(+)	Ortholog of Neosartorya fischeri NRRL 181 : NFIA_041190 and Aspergillus fumigatus A1163 : AFUB_049450
Afu5g01010	Chr5_A_fumigatus_Af293:261,795..262,839(+)	Ortholog of A. fumigatus Af293 : Afu8g00342, A. niger CBS 513.88 : An11g08170, Neosartorya fischeri NRRL 181 : NFIA_041160, NFIA_057730 and Aspergillus fumigatus A1163 : AFUB_049470, AFUB_086230
Afu5g01020	Chr5_A_fumigatus_Af293:263,220..264,401(+)	Ortholog(s) have N-terminal protein N-methyltransferase activity, role in N-terminal peptidyl-proline dimethylation, cytoplasmic translation, single-species biofilm formation on inanimate substrate and cytosol, nucleus localization
Afu5g01030	Chr5_A_fumigatus_Af293:266,294..270,326(-)	Putative glyceraldehyde 3-phosphate dehydrogenase
Afu5g01040	Chr5_A_fumigatus_Af293:271,173..271,976(+)	Ortholog(s) have versicolorin reductase activity and role in monodictyphenone biosynthetic process, sterigmatocystin biosynthetic process, xanthone-containing compound biosynthetic process
Afu5g01050	Chr5_A_fumigatus_Af293:273,337..274,032(+)	Ortholog of Aspergillus fumigatus A1163 : AFUB_049530
Afu5g01065	Chr5_A_fumigatus_Af293:274,190..276,403(-)	Has domain(s) with predicted DNA binding, RNA polymerase II transcription factor activity, sequence-specific DNA binding, zinc ion binding activity and role in regulation of transcription, DNA-templated, transcription, DNA-templated
Afu5g01080	Chr5_A_fumigatus_Af293:276,701..278,495(-)	Has domain(s) with predicted substrate-specific transmembrane transporter activity, transmembrane transporter activity, role in transmembrane transport and integral component of membrane, membrane localization
Afu5g01090	Chr5_A_fumigatus_Af293:279,017..280,663(+)	Has domain(s) with predicted ATP binding, phosphotransferase activity, alcohol group as acceptor activity and role in amino sugar metabolic process, peptidoglycan turnover
Afu5g01100	Chr5_A_fumigatus_Af293:281,249..282,903(+)	Ortholog of A. nidulans FGSC A4 : AN5259, A. fumigatus Af293 : Afu4g14660, A. niger CBS 513.88 : An16g07020, An02g11280 and Aspergillus wentii : Aspwe1_0055325, Aspwe1_0172784
Afu5g01110	Chr5_A_fumigatus_Af293:282,982..283,083(-)	Ortholog of Aspergillus fumigatus A1163 : AFUB_049590
Afu5g01120	Chr5_A_fumigatus_Af293:284,067..285,428(+)	Ortholog of A. nidulans FGSC A4 : AN8927, A. oryzae RIB40 : AO090003001298, Aspergillus flavus NRRL 3357 : AFL2T_01773 and Neosartorya fischeri NRRL 181 : NFIA_041050
Afu5g01130	Chr5_A_fumigatus_Af293:286,431..287,116(+)	Ortholog of Aspergillus fumigatus A1163 : AFUB_049610
Afu5g01140	Chr5_A_fumigatus_Af293:287,223..288,526(-)	Ortholog(s) have plasma membrane localization
Afu5g01160	Chr5_A_fumigatus_Af293:289,997..293,024(-)	Ortholog(s) have role in cellular response to drug and plasma membrane localization
Afu5g01170	Chr5_A_fumigatus_Af293:293,165..293,906(-)	Ortholog(s) have role in ethanol metabolic process and mitochondrial inner membrane localization
Afu5g01180	Chr5_A_fumigatus_Af293:294,359..295,991(-)	Has domain(s) with predicted GTP binding, GTPase activity and role in intracellular protein transport, nucleocytoplasmic transport, protein transport, signal transduction, small GTPase mediated signal transduction
Afu5g01190	Chr5_A_fumigatus_Af293:296,877..299,567(-)	Ortholog(s) have alpha-L-fucosidase activity and role in xyloglucan metabolic process
Afu5g01200	Chr5_A_fumigatus_Af293:299,899..301,571(-)	Putative carboxypeptidase S1
Afu5g01210	Chr5_A_fumigatus_Af293:302,633..304,030(-)	Ortholog(s) have extracellular region localization
Afu5g01220	Chr5_A_fumigatus_Af293:305,420..306,622(+)	Ortholog of Aspergillus wentii : Aspwe1_0024711 and Aspergillus fumigatus A1163 : AFUB_049690
Afu5g01230	Chr5_A_fumigatus_Af293:308,561..309,772(+)	RTA1 domain protein
Afu5g01240	Chr5_A_fumigatus_Af293:310,446..312,371(+)	Has domain(s) with predicted carbon-nitrogen ligase activity, with glutamine as amido-N-donor activity
Afu5g01242	Chr5_A_fumigatus_Af293:312,721..313,499(+)	Ortholog of Aspergillus fumigatus A1163 : AFUB_049720
Afu5g01245	Chr5_A_fumigatus_Af293:314,943..315,998(+)	Ortholog(s) have cytosol, nucleus localization
Afu5g01248	Chr5_A_fumigatus_Af293:316,147..317,731(+)	Ortholog of Aspergillus fumigatus A1163 : AFUB_049740
Afu5g01250	Chr5_A_fumigatus_Af293:318,205..319,062(+)	Has domain(s) with predicted coenzyme binding, oxidoreductase activity, acting on the CH-OH group of donors, NAD or NADP as acceptor, phosphogluconate dehydrogenase (decarboxylating) activity
Afu5g01260	Chr5_A_fumigatus_Af293:319,339..320,223(-)	Ortholog of A. oryzae RIB40 : AO090138000080, Aspergillus flavus NRRL 3357 : AFL2T_08758, Neosartorya fischeri NRRL 181 : NFIA_040860 and Aspergillus fumigatus A1163 : AFUB_049760
Afu5g01272	Chr5_A_fumigatus_Af293:322,314..324,173(+)	Has domain(s) with predicted DNA binding, RNA polymerase II transcription factor activity, sequence-specific DNA binding, zinc ion binding activity and role in regulation of transcription, DNA-templated, transcription, DNA-templated
Afu5g01290	Chr5_A_fumigatus_Af293:324,670..325,785(+)	Putative zinc-binding oxidoreductase
Afu5g01300	Chr5_A_fumigatus_Af293:326,045..327,838(-)	Ortholog of Neosartorya fischeri NRRL 181 : NFIA_040820, Aspergillus fumigatus A1163 : AFUB_049800 and Aspergillus clavatus NRRL 1 : ACLA_003970
Afu5g01310	Chr5_A_fumigatus_Af293:329,185..330,744(-)	Has domain(s) with predicted role in response to stress and integral component of membrane localization
Afu5g01320	Chr5_A_fumigatus_Af293:331,691..333,834(-)	Has domain(s) with predicted transmembrane transporter activity, role in transmembrane transport and integral component of membrane localization
Afu5g01330	Chr5_A_fumigatus_Af293:337,231..340,082(+)	Has domain(s) with predicted acid phosphatase activity, hydrolase activity, metal ion binding activity
Afu5g01340	Chr5_A_fumigatus_Af293:340,208..342,310(-)	Putative phospholipase B
Afu5g01350	Chr5_A_fumigatus_Af293:342,938..345,341(-)	Has domain(s) with predicted role in transmembrane transport and integral component of membrane localization
Afu5g01360	Chr5_A_fumigatus_Af293:345,827..351,157(+)	Ortholog(s) have oxidoreductase activity and role in steroid metabolic process
Afu5g01370	Chr5_A_fumigatus_Af293:351,293..352,418(-)	Ortholog of A. nidulans FGSC A4 : AN3630, Neosartorya fischeri NRRL 181 : NFIA_040730, Aspergillus versicolor : Aspve1_0032027 and Aspergillus fumigatus A1163 : AFUB_049880
Afu5g01380	Chr5_A_fumigatus_Af293:353,043..354,167(+)	Transcript up-regulated in conidia exposed to neutrophils
Afu5g01410	Chr5_A_fumigatus_Af293:369,915..373,565(+)	Has domain(s) with predicted ATP binding activity
Afu5g01420	Chr5_A_fumigatus_Af293:373,849..374,613(-)	Ortholog(s) have extracellular region localization
Afu5g01430	Chr5_A_fumigatus_Af293:375,182..376,001(-)	ThiJ/PfpI family protein
Afu5g01440	Chr5_A_fumigatus_Af293:376,504..377,687(-)	Ortholog(s) have thioredoxin peroxidase activity, role in cell redox homeostasis, cellular response to oxidative stress, response to metal ion and peroxisome, plasma membrane localization
Afu5g01450	Chr5_A_fumigatus_Af293:377,840..379,168(-)	Putative NADH-dependent flavin oxidoreductase
Afu5g01460	Chr5_A_fumigatus_Af293:379,479..381,009(-)	Has domain(s) with predicted RNA polymerase II transcription factor activity, sequence-specific DNA binding, zinc ion binding activity, role in regulation of transcription, DNA-templated and nucleus localization

Afu5g01470	Chr5_A_fumigatus_Af293:381,384..383,369(-)	Has domain(s) with predicted copper ion binding, primary amine oxidase activity, quinone binding activity and role in amine metabolic process, oxidation-reduction process
Afu5g01480	Chr5_A_fumigatus_Af293:383,966..385,567(+)	Ortholog of A. nidulans FGSC A4 : AN10795, AN6721, A. fumigatus Af293 : Afu2g13300, A. niger CBS 513.88 : An16g00890 and A. oryzae RIB40 : AO090011000070, AO090026000292
Afu5g01490	Chr5_A_fumigatus_Af293:386,888..388,165(-)	Putative hydrophobin
Afu5g01500	Chr5_A_fumigatus_Af293:388,348..389,425(+)	Homocysteine S-methyltransferase activity, role in L-methionine biosynthetic process from S-adenosylmethionine
Afu5g01510	Chr5_A_fumigatus_Af293:389,543..391,289(-)	Ortholog(s) have L-proline transmembrane transporter activity, role in proline transport and fungal-type vacuole, plasma membrane localization
Afu5g01520	Chr5_A_fumigatus_Af293:392,241..394,144(-)	Major facilitator superfamily protein
Afu5g01530	Chr5_A_fumigatus_Af293:395,030..396,105(-)	Has domain(s) with predicted oxidoreductase activity and role in metabolic process
Afu5g01540	Chr5_A_fumigatus_Af293:396,854..399,036(+)	Has domain(s) with predicted role in transmembrane transport and integral component of membrane localization
Afu5g01550	Chr5_A_fumigatus_Af293:399,752..402,216(+)	Ortholog(s) have role in hyphal growth, regulation of glycogen metabolic process
Afu5g01560	Chr5_A_fumigatus_Af293:402,386..402,604(-)	Ortholog of Aspergillus fumigatus A1163 : AFUB_050100
Afu5g01570	Chr5_A_fumigatus_Af293:402,779..403,792(-)	Ortholog of A. oryzae RIB40 : AO090011000267, Aspergillus glaucus : Aspgl1_0129601, Aspergillus flavus NRRL 3357 : AFL2T_05058 and Neosartorya fischeri NRRL 181 : NFIA_040510
Afu5g01580	Chr5_A_fumigatus_Af293:404,744..407,694(-)	Has domain(s) with predicted oxidoreductase activity and role in metabolic process
Afu5g01590	Chr5_A_fumigatus_Af293:407,927..409,582(+)	Has domain(s) with predicted role in isoprenoid biosynthetic process
Afu5g01600	Chr5_A_fumigatus_Af293:409,993..411,503(+)	Has domain(s) with predicted DNA binding, DNA-directed DNA polymerase activity, catalytic activity and role in DNA replication
Afu5g01610	Chr5_A_fumigatus_Af293:411,933..412,139(-)	Ortholog of Aspergillus fumigatus A1163 : AFUB_050150
Afu5g01620	Chr5_A_fumigatus_Af293:412,960..413,808(+)	Predicted adhesin-like protein
Afu5g01630	Chr5_A_fumigatus_Af293:414,816..416,800(+)	Ortholog(s) have drug transmembrane transporter activity and role in cellular response to biotic stimulus, drug transmembrane transport, fluconazole transport, peptide transport, spermidine transport
Afu5g01640	Chr5_A_fumigatus_Af293:417,463..418,617(+)	Ortholog of Neosartorya fischeri NRRL 181 : NFIA_040440, Aspergillus fumigatus A1163 : AFUB_050180 and Aspergillus clavatus NRRL 1 : ACLA_003640
Afu5g01650	Chr5_A_fumigatus_Af293:420,067..420,861(+)	Has domain(s) with predicted sequence-specific DNA binding, transcription factor activity, sequence-specific DNA binding activity and role in regulation of transcription, DNA-templated
Afu5g01662	Chr5_A_fumigatus_Af293:421,501..423,816(+)	Has domain(s) with predicted DNA binding, zinc ion binding activity, role in transcription, DNA-templated and nucleus localization
Afu5g01680	Chr5_A_fumigatus_Af293:423,967..425,737(-)	Ortholog(s) have endoplasmic reticulum localization
Afu5g01690	Chr5_A_fumigatus_Af293:426,111..427,078(+)	Ortholog(s) have cytosol, nucleus localization
Afu5g01700	Chr5_A_fumigatus_Af293:427,157..429,333(-)	Has domain(s) with predicted DNA binding, RNA polymerase II transcription factor activity, sequence-specific DNA binding, zinc ion binding activity and role in regulation of transcription, DNA-templated, transcription, DNA-templated
Afu5g01710	Chr5_A_fumigatus_Af293:429,669..431,519(-)	Cytochrome P450 phenylacetate 2-hydroxylase
Afu5g01720	Chr5_A_fumigatus_Af293:432,838..433,223(+)	Ortholog of Aspergillus fumigatus A1163 : AFUB_050250
Afu5g01730	Chr5_A_fumigatus_Af293:433,406..440,939(-)	Has domain(s) with predicted DNA binding, chromatin binding activity
Afu5g01740	Chr5_A_fumigatus_Af293:441,049..443,125(+)	Putative deoxyhypusine synthase
Afu5g01750	Chr5_A_fumigatus_Af293:443,187..448,026(+)	Has domain(s) with predicted ubiquitinyl hydrolase activity, zinc ion binding activity and role in protein deubiquitination, ubiquitin-dependent protein catabolic process
Afu5g01760	Chr5_A_fumigatus_Af293:448,246..450,547(-)	Ortholog(s) have rRNA (cytosine-C5-)-methyltransferase activity and role in assembly of large subunit precursor of preribosome, maturation of LSU-rRNA from tricistronic rRNA transcript (SSU-rRNA, 5.8S rRNA, LSU-rRNA), rRNA base methylation
Afu5g01770	Chr5_A_fumigatus_Af293:450,687..451,812(+)	Ortholog of A. nidulans FGSC A4 : AN8072, A. oryzae RIB40 : AO090003001347, Aspergillus wentii : Aspwe1_0029886, Aspergillus sydowii : Aspsy1_0156406 and Aspergillus terreus NIH2624 : ATET_09794
Afu5g01780	Chr5_A_fumigatus_Af293:452,401..455,057(-)	Ortholog(s) have role in ascospore formation, conidium formation, hyphal growth, regulation of ascospore formation, regulation of meiosis I and regulation of mitotic nuclear division, more
Afu5g01790	Chr5_A_fumigatus_Af293:455,314..455,541(-)	Predicted adhesin-like protein
Afu5g01800	Chr5_A_fumigatus_Af293:455,698..456,944(-)	DNA repair protein
Afu5g01810	Chr5_A_fumigatus_Af293:457,314..458,468(-)	Ortholog(s) have role in GPI anchor biosynthetic process
Afu5g01820	Chr5_A_fumigatus_Af293:458,980..463,583(+)	Ortholog(s) have role in Golgi to plasma membrane transport and fungal-type vacuole membrane localization
Afu5g01830	Chr5_A_fumigatus_Af293:463,789..466,012(-)	Has domain(s) with predicted catalytic activity, hydrolase activity, hydrolyzing O-glycosyl compounds activity and role in carbohydrate metabolic process
Afu5g01840	Chr5_A_fumigatus_Af293:466,591..470,453(-)	protein of unknown function
Afu5g01850	Chr5_A_fumigatus_Af293:470,727..472,720(+)	Ortholog(s) have tRNA (adenine-N1-)-methyltransferase activity, role in tRNA methylation and nucleus, tRNA (m1A) methyltransferase complex localization
Afu5g01860	Chr5_A_fumigatus_Af293:473,076..474,216(-)	Ortholog(s) have role in Arp2/3 complex-mediated actin nucleation, actin cortical patch localization, cellular response to drug, endocytosis and establishment of mitochondrion localization, more
Afu5g01870	Chr5_A_fumigatus_Af293:474,270..476,459(+)	Ortholog(s) have ATPase activity, DNA clamp loader activity and role in UV-damage excision repair, leading strand elongation, mitotic DNA damage checkpoint, sister chromatid cohesion
Afu5g01880	Chr5_A_fumigatus_Af293:476,542..478,803(+)	Has domain(s) with predicted acid phosphatase activity
Afu5g01890	Chr5_A_fumigatus_Af293:479,256..481,637(+)	Putative peptidyl-prolyl cis-trans isomerase
Afu5g01900	Chr5_A_fumigatus_Af293:482,209..485,134(-)	transcription factor
Afu5g01910	Chr5_A_fumigatus_Af293:485,997..487,013(-)	Ortholog of A. nidulans FGSC A4 : AN8036, A. niger CBS 513.88 : An16g01770, A. oryzae RIB40 : AO090003001328, Aspergillus wentii : Aspwe1_0042781 and Aspergillus sydowii : Aspsy1_0047861
Afu5g01920	Chr5_A_fumigatus_Af293:487,906..489,695(+)	Ortholog of A. niger CBS 513.88 : An16g01780, A. oryzae RIB40 : AO090003001326, Aspergillus wentii : Aspwe1_0174201, Aspergillus sydowii : Aspsy1_0029040 and Aspergillus terreus NIH2624 : ATET_09813
Afu5g01930	Chr5_A_fumigatus_Af293:489,700..491,868(-)	protein of unknown function
Afu5g01940	Chr5_A_fumigatus_Af293:493,699..498,757(+)	Has domain(s) with predicted nucleic acid binding, nucleotide binding activity
Afu5g01950	Chr5_A_fumigatus_Af293:498,909..500,329(-)	Ortholog(s) have chromatin binding activity
Afu5g01960	Chr5_A_fumigatus_Af293:501,133..503,280(+)	Ortholog(s) have role in phosphate ion transport, protein maturation and endoplasmic reticulum, mitochondrion, plasma membrane localization
Afu5g01970	Chr5_A_fumigatus_Af293:503,797..505,194(+)	glyceraldehyde-3-phosphate dehydrogenase
Afu5g01980	Chr5_A_fumigatus_Af293:505,597..510,023(+)	Major class 2 histone deacetylase
Afu5g01990	Chr5_A_fumigatus_Af293:511,005..511,475(+)	BYS1 domain protein
Afu5g02000	Chr5_A_fumigatus_Af293:511,853..514,101(-)	Has domain(s) with predicted proline racemase activity
Afu5g02010	Chr5_A_fumigatus_Af293:514,172..518,381(+)	Ortholog(s) have metalloendopeptidase activity
Afu5g02020	Chr5_A_fumigatus_Af293:519,080..520,101(+)	Putative aldehyde reductase
Afu5g02030	Chr5_A_fumigatus_Af293:520,484..521,754(+)	Has domain(s) with predicted mRNA binding activity, role in mRNA polyadenylation and mRNA cleavage factor complex localization
Afu5g02040	Chr5_A_fumigatus_Af293:522,625..524,528(+)	Putative extracellular lipase
Afu5g02050	Chr5_A_fumigatus_Af293:524,906..526,068(+)	Ortholog of A. nidulans FGSC A4 : AN11033, A. niger CBS 513.88 : An16g01890, A. oryzae RIB40 : AO090003001318, Aspergillus wentii : Aspwe1_0116157 and Aspergillus sydowii : Aspsy1_0207101
Afu5g02060	Chr5_A_fumigatus_Af293:526,816..527,634(+)	Ortholog(s) have role in coenzyme A biosynthetic process and CoA-synthesizing protein complex, endoplasmic reticulum, lipid particle, mitochondrion, nuclear envelope localization
Afu5g02070	Chr5_A_fumigatus_Af293:528,782..529,693(-)	Ortholog of A. nidulans FGSC A4 : AN8048, A. niger CBS 513.88 : An02g11210, A. oryzae RIB40 : AO090003001314, Aspergillus wentii : Aspwe1_0174218 and Aspergillus sydowii : Aspsy1_0155996
Afu5g02080	Chr5_A_fumigatus_Af293:529,805..530,664(+)	NADH-ubiquinone oxidoreductase with a role in oxidative phosphorylation
Afu5g02090	Chr5_A_fumigatus_Af293:531,007..531,795(-)	Ortholog(s) have cytosol, nucleus localization
Afu5g02100	Chr5_A_fumigatus_Af293:531,844..532,916(-)	Secreted protein of unknown function
Afu5g02110	Chr5_A_fumigatus_Af293:533,545..535,070(-)	Has domain(s) with predicted carbon-sulfur lyase activity and role in metabolic process
Afu5g02120	Chr5_A_fumigatus_Af293:535,310..538,537(+)	Ortholog of A. nidulans FGSC A4 : AN8051, A. niger CBS 513.88 : An02g11160, A. oryzae RIB40 : AO090003001306, Aspergillus wentii : Aspwe1_0029924 and Aspergillus sydowii : Aspsy1_0156226

Afu5g02130	Chr5_A_fumigatus_Af293:539,272..541,673(-)	Putative alpha-galactosidase
Afu5g02140	Chr5_A_fumigatus_Af293:541,877..547,180(-)	Putative inositol polyphosphate phosphatase
Afu5g02150	Chr5_A_fumigatus_Af293:547,635..548,545(-)	Putative proteasome component
Afu5g02160	Chr5_A_fumigatus_Af293:548,997..552,615(-)	Ortholog(s) have nucleolus localization
Afu5g02170	Chr5_A_fumigatus_Af293:554,444..558,690(-)	Ortholog(s) have ATPase activity, DNA/DNA annealing activity, role in mitotic chromosome condensation and cytosol, nuclear condensin complex localization
Afu5g02180	Chr5_A_fumigatus_Af293:559,007..560,286(+)	Cysteine synthase (o-acetylserine (thiol)-lyase)
Afu5g02190	Chr5_A_fumigatus_Af293:560,398..561,745(-)	Has domain(s) with predicted electron carrier activity, metal ion binding, molybdenum ion binding, oxidoreductase activity and role in nitrate assimilation, oxidation-reduction process
Afu5g02200	Chr5_A_fumigatus_Af293:561,979..563,147(-)	Ortholog(s) have mitochondrion targeting sequence binding, protein channel activity, role in protein import into mitochondrial inner membrane and mitochondrial inner membrane protein insertion complex, plasma membrane localization
Afu5g02210	Chr5_A_fumigatus_Af293:563,258..565,725(-)	Ortholog of A. nidulans FGSC A4 : AN8034, A. niger CBS 513.88 : An02g10730, A. oryzae RIB40 : AO090102000294, Aspergillus wentii : Aspwe1_0114775 and Aspergillus sydowii : Aspsy1_0033693
Afu5g02220	Chr5_A_fumigatus_Af293:566,033..569,100(-)	Ortholog(s) have role in ascospore formation, conidiophore development, conidium formation, hyphal growth, regulation of protein localization to mitotic spindle pole body and septation initiation signaling, more
Afu5g02230	Chr5_A_fumigatus_Af293:569,279..571,748(-)	Putative actin interacting protein
Afu5g02240	Chr5_A_fumigatus_Af293:571,955..573,035(+)	NAD dependent epimerase/dehydratase family protein
Afu5g02250	Chr5_A_fumigatus_Af293:574,480..577,620(+)	Ortholog of A. nidulans FGSC A4 : AN8031, A. niger CBS 513.88 : An02g10580, A. oryzae RIB40 : AO090102000288, Aspergillus wentii : Aspwe1_0042830 and Aspergillus sydowii : Aspsy1_0156829
Afu5g02260	Chr5_A_fumigatus_Af293:577,630..582,809(-)	Putative ABC multidrug transporter
Afu5g02270	Chr5_A_fumigatus_Af293:583,247..585,836(+)	Fungal specific transcription factor domain family protein
Afu5g02280	Chr5_A_fumigatus_Af293:586,719..588,210(-)	Has domain(s) with predicted hydrolase activity, hydrolyzing O-glycosyl compounds activity and role in carbohydrate metabolic process
Afu5g02290	Chr5_A_fumigatus_Af293:589,449..592,756(-)	Ortholog(s) have chloride transmembrane transporter activity, potassium uptake transmembrane transporter activity
Afu5g02300	Chr5_A_fumigatus_Af293:593,925..595,013(-)	Has domain(s) with predicted peroxidase activity
Afu5g02310	Chr5_A_fumigatus_Af293:595,247..596,570(-)	Ortholog of A. nidulans FGSC A4 : AN8028, A. niger CBS 513.88 : An02g10470, A. oryzae RIB40 : AO090102000345, Aspergillus sydowii : Aspsy1_0059896 and Aspergillus terreus NIH2624 : ATET_09200
Afu5g02320	Chr5_A_fumigatus_Af293:596,583..597,904(-)	Secreted protein of unknown function
Afu5g02330	Chr5_A_fumigatus_Af293:598,210..599,272(+)	Allergen Asp f 1
Afu5g02340	Chr5_A_fumigatus_Af293:600,037..603,026(+)	Ortholog of A. nidulans FGSC A4 : AN8027, A. niger CBS 513.88 : An09g03700, Neosartorya fischeri NRRL 181 : NFIA_039750, Aspergillus versicolor : Aspve1_0154183 and Aspergillus zonatus : Aspzo1_0013765
Afu5g02350	Chr5_A_fumigatus_Af293:603,109..604,368(+)	Putative carbon-nitrogen family hydrolase
Afu5g02360	Chr5_A_fumigatus_Af293:605,751..609,032(-)	Ortholog(s) have role in hyphal growth, peroxisome organization, vacuole organization and Golgi apparatus localization
Afu5g02370	Chr5_A_fumigatus_Af293:609,205..611,860(-)	Vacuolar ATP synthase catalytic subunit A
Afu5g02380	Chr5_A_fumigatus_Af293:612,705..614,436(+)	Ortholog of A. nidulans FGSC A4 : AN8020, AN0195, A. niger CBS 513.88 : An03g04900, An02g10420, A. oryzae RIB40 : AO090001000067, AO090102000350 and Aspergillus wentii : Aspwe1_0114436, Aspwe1_0176498
Afu5g02390	Chr5_A_fumigatus_Af293:614,621..617,783(+)	Has domain(s) with predicted role in transmembrane transport and integral component of membrane localization
Afu5g02400	Chr5_A_fumigatus_Af293:617,914..620,924(+)	Ortholog(s) have cytosol, mitotic spindle pole body, nucleus localization
Afu5g02410	Chr5_A_fumigatus_Af293:621,886..623,486(-)	Putative DEAD/DEAH box helicase
Afu5g02420	Chr5_A_fumigatus_Af293:623,488..625,330(+)	Ortholog(s) have U2 snRNP, cytosol localization
Afu5g02430	Chr5_A_fumigatus_Af293:625,685..628,600(+)	Has domain(s) with predicted ATP binding, DNA binding, DNA ligase (ATP) activity and role in DNA recombination, DNA repair
Afu5g02440	Chr5_A_fumigatus_Af293:629,040..631,759(-)	Ortholog(s) have role in positive regulation of mitotic metaphase/anaphase transition and anaphase-promoting complex, mitotic spindle pole body localization
Afu5g02450	Chr5_A_fumigatus_Af293:631,975..633,093(+)	Putative polyprenyl synthetase
Afu5g02460	Chr5_A_fumigatus_Af293:634,677..636,602(+)	Ortholog of A. nidulans FGSC A4 : AN5465, A. niger CBS 513.88 : An02g10330, An07g05030, A. oryzae RIB40 : AO090102000359, Aspergillus wentii : Aspwe1_0114453 and Aspergillus sydowii : Aspsy1_0156722
Afu5g02470	Chr5_A_fumigatus_Af293:636,920..638,606(-)	Thiamine biosynthesis protein, phoB-regulated
Afu5g02480	Chr5_A_fumigatus_Af293:639,550..641,874(-)	Putative glycogen sythase
Afu5g02490	Chr5_A_fumigatus_Af293:643,973..644,968(-)	Has domain(s) with predicted oxidoreductase activity, transferase activity, transferring acyl groups other than amino-acyl groups, zinc ion binding activity and role in oxidation-reduction process
Afu5g02500	Chr5_A_fumigatus_Af293:645,430..646,082(+)	protein of unknown function
Afu5g02510	Chr5_A_fumigatus_Af293:646,444..650,093(-)	Ortholog(s) have role in Golgi to vacuole transport, ascospore wall assembly, retrograde transport, endosome to Golgi and GARP complex, mitochondrion localization
Afu5g02520	Chr5_A_fumigatus_Af293:650,260..652,927(+)	Ortholog(s) have DNA replication origin binding, chromatin binding, single-stranded DNA binding, single-stranded DNA-dependent ATP-dependent DNA helicase activity
Afu5g02530	Chr5_A_fumigatus_Af293:653,089..654,944(+)	Has domain(s) with predicted ribokinase activity and role in D-ribose metabolic process
Afu5g02540	Chr5_A_fumigatus_Af293:655,928..657,939(+)	Ortholog of A. nidulans FGSC A4 : AN7996, A. niger CBS 513.88 : An02g10230, A. oryzae RIB40 : AO090102000369, Aspergillus wentii : Aspwe1_0029974 and Aspergillus sydowii : Aspsy1_0156606
Afu5g02550	Chr5_A_fumigatus_Af293:658,329..660,917(-)	Ortholog(s) have chromatin insulator sequence binding, transcription factor activity, core RNA polymerase III binding activity and role in transcription initiation from RNA polymerase III promoter
Afu5g02560	Chr5_A_fumigatus_Af293:661,646..665,087(+)	Ortholog(s) have protein phosphatase type 2A regulator activity
Afu5g02570	Chr5_A_fumigatus_Af293:665,117..677,364(+)	Ortholog(s) have histone acetyltransferase activity
Afu5g02580	Chr5_A_fumigatus_Af293:677,833..678,957(-)	Ortholog(s) have U2-type spliceosomal complex, mitotic spindle pole body localization
Afu5g02590	Chr5_A_fumigatus_Af293:679,022..682,214(+)	Ortholog(s) have role in positive regulation of mitotic metaphase/anaphase transition and anaphase-promoting complex, cytosol localization
Afu5g02600	Chr5_A_fumigatus_Af293:683,315..684,766(+)	Ortholog of A. nidulans FGSC A4 : AN8003, A. niger CBS 513.88 : An02g10100, Aspergillus wentii : Aspwe1_0174280, Aspergillus sydowii : Aspsy1_0033725 and Aspergillus terreus NIH2624 : ATET_09228
Afu5g02610	Chr5_A_fumigatus_Af293:685,654..687,128(+)	Has domain(s) with predicted electron carrier activity, heme binding, iron ion binding, oxidoreductase activity, acting on paired donors, with incorporation or reduction of molecular oxygen activity and role in oxidation-reduction process
Afu5g02620	Chr5_A_fumigatus_Af293:687,330..689,013(-)	Ortholog(s) have oxidoreductase activity, oxygen binding activity and role in ascospore wall assembly, chlamydospore formation
Afu5g02630	Chr5_A_fumigatus_Af293:689,239..689,978(+)	Ortholog of Neosartorya fischeri NRRL 181 : NFIA_039450, Aspergillus fumigatus A1163 : AFUB_051160, Aspergillus clavatus NRRL 1 : ACLA_002530 and Aspergillus terreus NIH2624 : ATET_09232
Afu5g02640	Chr5_A_fumigatus_Af293:690,252..692,134(+)	Has domain(s) with predicted O-methyltransferase activity
Afu5g02650	Chr5_A_fumigatus_Af293:692,297..693,109(-)	protein of unknown function
Afu5g02655	Chr5_A_fumigatus_Af293:693,142..696,385(+)	Has domain(s) with predicted sequence-specific DNA binding, transcription factor activity, sequence-specific DNA binding activity and role in regulation of transcription, DNA-templated
Afu5g02660	Chr5_A_fumigatus_Af293:696,501..699,185(-)	Has domain(s) with predicted oxidoreductase activity and role in oxidation-reduction process
Afu5g02670	Chr5_A_fumigatus_Af293:700,135..700,845(-)	Ortholog of A. nidulans FGSC A4 : AN6760, AN8527, A. fumigatus Af293 : Afu4g01400, Afu5g14240, A. niger CBS 513.88 : An07g09050, An08g09630 and Aspergillus wentii : Aspwe1_0069899, Aspwe1_0185315, Aspwe1_0186212
Afu5g02690	Chr5_A_fumigatus_Af293:702,770..705,415(-)	Ortholog(s) have role in conidiophore development
Afu5g02700	Chr5_A_fumigatus_Af293:706,165..707,944(-)	Putative multidrug resistant protein
Afu5g02710	Chr5_A_fumigatus_Af293:708,150..708,374(+)	protein of unknown function
Afu5g02720	Chr5_A_fumigatus_Af293:710,324..714,412(+)	Ortholog(s) have phosphoribosylformylglycinamidine synthase activity, role in purine nucleotide biosynthetic process and Golgi apparatus localization
Afu5g02730	Chr5_A_fumigatus_Af293:714,808..717,864(-)	Ortholog(s) have gamma-tubulin binding activity and spindle pole body localization
Afu5g02740	Chr5_A_fumigatus_Af293:717,912..719,315(+)	Putative alpha-1,2-mannosyltransferase with a predicted role in N-linked protein glycosylation
Afu5g02750	Chr5_A_fumigatus_Af293:719,993..720,857(-)	Cytochrome c oxidase subunit Va with a predicted role in oxidative phosphorylation
Afu5g02760	Chr5_A_fumigatus_Af293:722,123..724,776(+)	Putative fatty acid elongase with a predicted role in fatty acid biosynthesis
Afu5g02770	Chr5_A_fumigatus_Af293:727,468..731,528(+)	Ortholog of A. nidulans FGSC A4 : AN8116, A. niger CBS 513.88 : An02g09830, A. oryzae RIB40 : AO090102000396, Aspergillus wentii : Aspwe1_0029998 and Aspergillus sydowii : Aspsy1_0050460

Afu5g02780	Chr5_A_fumigatus_Af293:732,988..737,296(+)	Putative mitochondrial nicotinamide nucleotide transhydrogenase subunit
Afu5g02785	Chr5_A_fumigatus_Af293:737,337..738,299(-)	Ortholog of Neosartorya fischeri NRRL 181 : NFIA_039310 and Aspergillus fumigatus A1163 : AFUB_051320
Afu5g02790	Chr5_A_fumigatus_Af293:739,720..742,121(+)	Ortholog(s) have thiamine transmembrane transporter activity, role in thiamine transmembrane transport and endoplasmic reticulum, medial membrane band, plasma membrane of cell tip localization
Afu5g02800	Chr5_A_fumigatus_Af293:742,624..744,489(-)	C6 transcription factor
Afu5g02820	Chr5_A_fumigatus_Af293:749,896..750,562(-)	Ortholog of Aspergillus glaucus : Aspgl1_0049726, Neosartorya fischeri NRRL 181 : NFIA_039280, Aspergillus fumigatus A1163 : AFUB_051350 and Aspergillus clavatus NRRL 1 : ACLA_002340
Afu5g02840	Chr5_A_fumigatus_Af293:750,813..753,776(-)	Has domain(s) with predicted substrate-specific transmembrane transporter activity, transmembrane transporter activity, role in transmembrane transport and integral component of membrane, membrane localization
Afu5g02850	Chr5_A_fumigatus_Af293:754,271..758,025(+)	Has domain(s) with predicted arylformamidase activity and role in tryptophan catabolic process to kynurenine
Afu5g02860	Chr5_A_fumigatus_Af293:758,332..760,220(+)	Ortholog of A. nidulans FGSC A4 : AN2683, AN5069, A. fumigatus Af293 : Afu5g14320, A. niger CBS 513.88 : An09g04830, An14g05730 and A. oryzae RIB40 : AO090102000482, AO090001000197
Afu5g02870	Chr5_A_fumigatus_Af293:760,729..762,034(+)	Has domain(s) with predicted oxidoreductase activity and role in metabolic process
Afu5g02880	Chr5_A_fumigatus_Af293:762,083..764,748(-)	Ortholog(s) have nucleic acid binding transcription factor activity
Afu5g02890	Chr5_A_fumigatus_Af293:764,921..767,461(-)	Ortholog of A. nidulans FGSC A4 : AN8165, A. niger CBS 513.88 : An09g04870, A. oryzae RIB40 : AO090102000485 and Aspergillus wentii : Aspwe1_0042540, Aspwe1_0042989
Afu5g02900	Chr5_A_fumigatus_Af293:769,794..770,829(+)	Ortholog(s) have Golgi apparatus, cell septum, endoplasmic reticulum localization
Afu5g02910	Chr5_A_fumigatus_Af293:770,881..772,556(-)	NAP family protein
Afu5g02920	Chr5_A_fumigatus_Af293:773,874..774,997(-)	Ortholog(s) have NAD+ binding, sequence-specific DNA binding activity, role in nitrogen catabolite repression of transcription from RNA polymerase II promoter, regulation of nitrate assimilation and nucleus localization
Afu5g02930	Chr5_A_fumigatus_Af293:778,620..780,409(-)	Has domain(s) with predicted role in cellular amino acid metabolic process
Afu5g02940	Chr5_A_fumigatus_Af293:781,599..783,300(-)	Ortholog(s) have (R)-carnitine transmembrane transporter activity, choline transmembrane transporter activity, ethanolamine transmembrane transporter activity
Afu5g02950	Chr5_A_fumigatus_Af293:786,805..790,619(+)	Ortholog of A. nidulans FGSC A4 : AN8167, A. niger CBS 513.88 : An09g05040, A. oryzae RIB40 : AO090102000494, Aspergillus wentii : Aspwe1_0030018 and Aspergillus sydowii : Aspsy1_0061808
Afu5g02960	Chr5_A_fumigatus_Af293:790,739..791,184(-)	Ortholog of Aspergillus fumigatus A1163 : AFUB_051480
Afu5g02970	Chr5_A_fumigatus_Af293:792,199..794,897(-)	Ortholog of A. nidulans FGSC A4 : AN12172, A. niger CBS 513.88 : An09g02540, A. oryzae RIB40 : AO090102000497, Aspergillus wentii : Aspwe1_0114955 and Aspergillus sydowii : Aspsy1_0161769
Afu5g02990	Chr5_A_fumigatus_Af293:797,458..799,870(+)	Putative aromatic amino acid aminotransferase
Afu5g03000	Chr5_A_fumigatus_Af293:799,891..801,824(+)	Ortholog(s) have actin monomer binding, protein kinase inhibitor activity, ribosome binding activity, role in negative regulation of protein phosphorylation and cytoplasm, nucleus, polysome, ribosome localization
Afu5g03010	Chr5_A_fumigatus_Af293:803,554..806,502(+)	protein of unknown function
Afu5g03020	Chr5_A_fumigatus_Af293:806,575..808,149(+)	60S ribosomal protein L4
Afu5g03030	Chr5_A_fumigatus_Af293:814,229..819,740(+)	Has domain(s) with predicted DNA binding, RNA polymerase II transcription factor activity, sequence-specific DNA binding, zinc ion binding activity and role in regulation of transcription, DNA-templated, transcription, DNA-templated
Afu5g03040	Chr5_A_fumigatus_Af293:820,242..823,363(+)	Ortholog of A. nidulans FGSC A4 : AN8179, A. oryzae RIB40 : AO090102000505, Aspergillus wentii : Aspwe1_0053028, Aspergillus sydowii : Aspsy1_0050623 and Aspergillus terreus NIH2624 : ATET_09281
Afu5g03050	Chr5_A_fumigatus_Af293:823,511..827,029(+)	Ortholog(s) have role in mRNA cis splicing, via spliceosome and Prp19 complex, mitotic spindle pole body, spliceosomal complex localization
Afu5g03060	Chr5_A_fumigatus_Af293:828,603..830,127(+)	Ortholog of A. nidulans FGSC A4 : AN8181, A. niger CBS 513.88 : An09g05240, A. oryzae RIB40 : AO090102000509, Aspergillus wentii : Aspwe1_0053030 and Aspergillus sydowii : Aspsy1_0050627

Gene ID	Genomic Location (Gene)	Product Description
Afu5g12720	Chr5_A_fumigatus_Af293:3,307,809..3,313,231(-)	Putative ABC multidrug transporter
Afu5g12730	Chr5_A_fumigatus_Af293:3,314,537..3,340,664(+)	Nonribosomal peptide synthetase 8
Afu5g12740	Chr5_A_fumigatus_Af293:3,340,680..3,343,467(+)	Putative MFS multidrug transporter
Afu5g12750	Chr5_A_fumigatus_Af293:3,343,555..3,346,198(-)	hypothetical protein
Afu5g12760	Chr5_A_fumigatus_Af293:3,346,707..3,348,654(-)	CCCH zinc finger DNA binding protein
Afu5g12770	Chr5_A_fumigatus_Af293:3,349,005..3,350,331(-)	metallo-beta-lactamase superfamily protein
Afu5g12780	Chr5_A_fumigatus_Af293:3,350,380..3,353,129(+)	hypothetical protein
Afu5g12790	Chr5_A_fumigatus_Af293:3,353,314..3,355,167(-)	mitochondrial 3-hydroxyisobutyryl-CoA hydrolase, putative
Afu5g12800	Chr5_A_fumigatus_Af293:3,355,297..3,356,439(-)	Ortholog(s) have structural constituent of ribosome activity and mitochondrial large ribosomal subunit localization
Afu5g12810	Chr5_A_fumigatus_Af293:3,356,560..3,357,895(+)	Ortholog(s) have structural constituent of ribosome activity and mitochondrial large ribosomal subunit localization
Afu5g12820	Chr5_A_fumigatus_Af293:3,358,074..3,358,750(-)	Ortholog(s) have endopeptidase activity, role in protein processing involved in protein targeting to mitochondrion and mitochondrial inner membrane peptidase complex localization
Afu5g12830	Chr5_A_fumigatus_Af293:3,358,866..3,360,837(-)	Ortholog of A. nidulans FGSC A4 : AN7959, A. oryzae RIB40 : AO090120000409, Neosartorya fischeri NRRL 181 : NFIA_074340, Aspergillus wentii : Aspwe1_0449236 and Aspergillus versicolor : Aspve1_0175438
Afu5g12840	Chr5_A_fumigatus_Af293:3,361,748..3,362,965(-)	Putative hydroxyacylglutathione hydrolase
Afu5g12850	Chr5_A_fumigatus_Af293:3,363,320..3,364,625(-)	Ortholog of A. nidulans FGSC A4 : AN6822, A. niger CBS 513.88 : An14g06350, A. oryzae RIB40 : AO090120000405, Neosartorya fischeri NRRL 181 : NFIA_074310 and Aspergillus wentii : Aspwe1_0026733
Afu5g12860	Chr5_A_fumigatus_Af293:3,365,321..3,367,316(-)	Ortholog of A. nidulans FGSC A4 : AN6823, A. niger CBS 513.88 : An14g06360, A. oryzae RIB40 : AO090120000406, Aspergillus wentii : Aspwe1_0039015 and Aspergillus sydowii : Aspsy1_0142898
Afu5g12870	Chr5_A_fumigatus_Af293:3,375,118..3,377,520(+)	Ortholog(s) have cytosol, nucleus localization
Afu5g12880	Chr5_A_fumigatus_Af293:3,378,475..3,379,503(+)	Ortholog(s) have Rab guanyl-nucleotide exchange factor activity, role in ER to Golgi vesicle-mediated transport and TRAPPII protein complex, cytosol, nuclear envelope localization
Afu5g12890	Chr5_A_fumigatus_Af293:3,379,940..3,381,478(-)	Ortholog of A. nidulans FGSC A4 : AN6826, A. niger CBS 513.88 : An14g06450, A. oryzae RIB40 : AO090120000402, Aspergillus wentii : Aspwe1_0058891 and Aspergillus sydowii : Aspsy1_0143996
Afu5g12895	Chr5_A_fumigatus_Af293:3,381,871..3,382,377(+)	Ortholog(s) have role in posttranscriptional gene silencing, production of small RNA involved in gene silencing by RNA
Afu5g12900	Chr5_A_fumigatus_Af293:3,383,154..3,386,795(-)	Has domain(s) with predicted sequence-specific DNA binding, transcription factor activity, sequence-specific DNA binding, zinc ion binding activity and role in regulation of transcription, DNA-templated
Afu5g12910	Chr5_A_fumigatus_Af293:3,387,547..3,389,130(+)	Ortholog(s) have role in mRNA splicing, via spliceosome and Prp19 complex, U1 snRNP, U2 snRNP, U2-type prespliceosome, U4/U6 x U5 tri-snRNP complex, U5 snRNP localization
Afu5g12920	Chr5_A_fumigatus_Af293:3,389,488..3,391,310(+)	Putative iron-regulated transporter
Afu5g12930	Chr5_A_fumigatus_Af293:3,391,693..3,393,741(+)	Has domain(s) with predicted RNA polymerase II transcription factor activity, sequence-specific DNA binding, zinc ion binding activity, role in regulation of transcription, DNA-templated and nucleus localization
Afu5g12940	Chr5_A_fumigatus_Af293:3,393,897..3,396,049(-)	Ortholog(s) have cytosol, nucleus localization
Afu5g12950	Chr5_A_fumigatus_Af293:3,396,529..3,398,512(+)	Has domain(s) with predicted substrate-specific transmembrane transporter activity, transmembrane transporter activity, role in transmembrane transport and integral component of membrane, membrane localization
Afu5g12960	Chr5_A_fumigatus_Af293:3,398,724..3,399,710(-)	Putative bZip-type transcription factor with similarity to atfA
Afu5g12970	Chr5_A_fumigatus_Af293:3,400,893..3,401,310(-)	protein of unknown function
Afu5g12980	Chr5_A_fumigatus_Af293:3,403,146..3,404,152(-)	Ortholog of Aspergillus glaucus : Aspgl1_0105053 and Aspergillus fumigatus A1163 : AFUB_060700
Afu5g12990	Chr5_A_fumigatus_Af293:3,406,140..3,408,009(-)	Snf1 protein kinase complex subunit
Afu5g13000	Chr5_A_fumigatus_Af293:3,408,677..3,410,548(-)	Ortholog(s) have phosphatidylinositol transporter activity
Afu5g13020	Chr5_A_fumigatus_Af293:3,410,658..3,412,898(+)	Ortholog(s) have DNA-directed DNA polymerase activity, role in DNA replication initiation, telomere capping and alpha DNA polymerase:primase complex, cytosol, nuclear envelope localization
Afu5g13030	Chr5_A_fumigatus_Af293:3,413,073..3,413,860(-)	Ortholog of A. nidulans FGSC A4 : AN6854, A. oryzae RIB40 : AO090120000423, Neosartorya fischeri NRRL 181 : NFIA_074070, Aspergillus wentii : Aspwe1_0026749 and Aspergillus versicolor : Aspve1_0211277
Afu5g13040	Chr5_A_fumigatus_Af293:3,414,201..3,415,930(-)	Ortholog(s) have enzyme regulator activity, guanyl-nucleotide exchange factor activity, translation initiation factor activity and role in regulation of translational initiation
Afu5g13050	Chr5_A_fumigatus_Af293:3,416,535..3,418,950(-)	Ortholog(s) have microtubule motor activity, protein homodimerization activity and cell tip, kinesin complex, medial cortical node, mitotic spindle pole body, nucleus localization
Afu5g13060	Chr5_A_fumigatus_Af293:3,421,138..3,422,353(+)	Ortholog of A. nidulans FGSC A4 : AN6861/phbB, AN9483, A. fumigatus Af293 : Afu3g00670, Afu8g01120, A. niger CBS 513.88 : An03g01130, An07g06330, An07g00905 and A. oryzae RIB40 : AO090120000428, AO090113000089
Afu5g13070	Chr5_A_fumigatus_Af293:3,423,600..3,425,578(-)	Ortholog of A. nidulans FGSC A4 : AN6859, A. niger CBS 513.88 : An14g06090, A. oryzae RIB40 : AO090120000432, Aspergillus wentii : Aspwe1_0107789 and Aspergillus sydowii : Aspsy1_0085559
Afu5g13080	Chr5_A_fumigatus_Af293:3,430,092..3,434,136(-)	Has domain(s) with predicted RNA polymerase II transcription factor activity, sequence-specific DNA binding, zinc ion binding activity, role in regulation of transcription, DNA-templated and nucleus localization
Afu5g13090	Chr5_A_fumigatus_Af293:3,437,488..3,438,966(+)	Has domain(s) with predicted transferase activity, transferring glycosyl groups activity and role in protein glycosylation
Afu5g13100	Chr5_A_fumigatus_Af293:3,439,399..3,439,899(-)	Ortholog of A. nidulans FGSC A4 : AN6856, A. niger CBS 513.88 : An14g06050, A. oryzae RIB40 : AO090120000435, Aspergillus wentii : Aspwe1_0107169 and Aspergillus sydowii : Aspsy1_0040420
Afu5g13110	Chr5_A_fumigatus_Af293:3,440,556..3,441,269(+)	Ortholog(s) have cytosol, nucleus localization
Afu5g13120	Chr5_A_fumigatus_Af293:3,441,589..3,442,686(+)	Ortholog(s) have endoplasmic reticulum, nuclear envelope localization
Afu5g13130	Chr5_A_fumigatus_Af293:3,442,869..3,443,857(-)	Putative chorismate mutase
Afu5g13140	Chr5_A_fumigatus_Af293:3,444,254..3,447,140(+)	Ortholog(s) have ubiquitin binding activity, role in mitochondrial fission, mitochondrial genome maintenance, peroxisome fission and mitochondrial outer membrane localization
Afu5g13150	Chr5_A_fumigatus_Af293:3,447,599..3,450,836(+)	Ortholog of A. nidulans FGSC A4 : AN6868, A. niger CBS 513.88 : An14g05990, A. oryzae RIB40 : AO090120000440, Aspergillus wentii : Aspwe1_0050582 and Aspergillus sydowii : Aspsy1_0054814
Afu5g13160	Chr5_A_fumigatus_Af293:3,452,444..3,455,043(-)	Has domain(s) with predicted role in transmembrane transport and integral component of membrane localization
Afu5g13170	Chr5_A_fumigatus_Af293:3,455,726..3,457,692(+)	Putative MATE efflux family protein
Afu5g13180	Chr5_A_fumigatus_Af293:3,458,782..3,460,652(+)	Putative agmatinase, phoB-regulated
Afu5g13190	Chr5_A_fumigatus_Af293:3,462,822..3,469,726(+)	Has domain(s) with predicted ATP binding, microtubule binding, microtubule motor activity, role in microtubule-based movement and microtubule associated complex localization
Afu5g13200	Chr5_A_fumigatus_Af293:3,469,967..3,470,629(+)	Ortholog of A. nidulans FGSC A4 : AN4261, AN12307, A. fumigatus Af293 : Afu7g03960, A. oryzae RIB40 : AO090020000704, AO090026000045, AO090026000818 and Aspergillus wentii : Aspwe1_0181783
Afu5g13210	Chr5_A_fumigatus_Af293:3,470,836..3,472,312(+)	Ortholog(s) have GDP-Man:Man1GlcNAc2-PP-Dol alpha-1,3-mannosyltransferase activity, glycolipid 6-alpha-mannosyltransferase activity and role in oligosaccharide-lipid intermediate biosynthetic process
Afu5g13220	Chr5_A_fumigatus_Af293:3,472,524..3,474,541(+)	Ortholog(s) have crossover junction endodeoxyribonuclease activity and role in DNA repair, DNA topological change, regulation of reciprocal meiotic recombination, resolution of meiotic recombination intermediates
Afu5g13230	Chr5_A_fumigatus_Af293:3,474,874..3,477,195(-)	Ortholog(s) have fungal-type vacuole membrane localization
Afu5g13240	Chr5_A_fumigatus_Af293:3,477,213..3,482,789(-)	Has domain(s) with predicted inositol-1,4,5-trisphosphate 3-kinase activity
Afu5g13250	Chr5_A_fumigatus_Af293:3,485,112..3,487,485(+)	Ortholog of A. nidulans FGSC A4 : AN6885, A. niger CBS 513.88 : An14g04640, A. oryzae RIB40 : AO090120000468, Aspergillus sydowii : Aspsy1_0054799 and Aspergillus terreus NIH2624 : ATET_06186
Afu5g13260	Chr5_A_fumigatus_Af293:3,488,403..3,488,757(-)	Ortholog of Aspergillus fumigatus A1163 : AFUB_060970
Afu5g13270	Chr5_A_fumigatus_Af293:3,488,865..3,491,687(+)	pH-response regulator protein
Afu5g13280	Chr5_A_fumigatus_Af293:3,491,768..3,494,412(+)	Ortholog of A. nidulans FGSC A4 : AN6887, A. niger CBS 513.88 : An14g04670, A. oryzae RIB40 : AO090120000470, Aspergillus wentii : Aspwe1_0039072 and Aspergillus sydowii : Aspsy1_0145002
Afu5g13290	Chr5_A_fumigatus_Af293:3,494,584..3,496,310(-)	Has domain(s) with predicted role in transmembrane transport and integral component of membrane localization
Afu5g13300	Chr5_A_fumigatus_Af293:3,496,895..3,499,105(-)	Putative extracellular aspartic endopeptidase
Afu5g13310	Chr5_A_fumigatus_Af293:3,500,569..3,505,002(-)	Ortholog(s) have RNA polymerase II transcription factor activity, sequence-specific DNA binding, sequence-specific DNA binding activity
Afu5g13320	Chr5_A_fumigatus_Af293:3,505,018..3,507,277(-)	Ortholog(s) have cytosol localization
Afu5g13330	Chr5_A_fumigatus_Af293:3,507,706..3,508,803(+)	Ortholog of A. nidulans FGSC A4 : AN6891, A. niger CBS 513.88 : An14g04740, A. oryzae RIB40 : AO090120000478, Aspergillus wentii : Aspwe1_0050596 and Aspergillus sydowii : Aspsy1_0085527
Afu5g13340	Chr5_A_fumigatus_Af293:3,509,452..3,511,837(-)	Ortholog(s) have magnesium-dependent protein serine/threonine phosphatase activity
Afu5g13350	Chr5_A_fumigatus_Af293:3,512,436..3,514,578(-)	Putative peptidyl-prolyl cis-trans isomerase

Afu5g13360	Chr5_A_fumigatus_Af293:3,514,875..3,517,467(-)	Ortholog(s) have mRNA binding activity
Afu5g13370	Chr5_A_fumigatus_Af293:3,517,576..3,520,528(-)	Ortholog(s) have ATP-dependent 5'-3' DNA helicase activity, single-stranded DNA-dependent ATP-dependent DNA helicase activity
Afu5g13380	Chr5_A_fumigatus_Af293:3,521,064..3,524,202(+)	Ortholog(s) have protein complex scaffold activity and role in mitotic DNA damage checkpoint, mitotic DNA replication checkpoint, mitotic DNA replication preinitiation complex assembly
Afu5g13392	Chr5_A_fumigatus_Af293:3,524,528..3,527,656(-)	Ortholog(s) have GTPase activity, protein homodimerization activity, role in mitochondrial fusion, mitochondrion localization and integral component of mitochondrial outer membrane, mitochondrial inner membrane localization
Afu5g13410	Chr5_A_fumigatus_Af293:3,527,728..3,529,048(+)	Ortholog(s) have role in asexual sporulation resulting in formation of a cellular spore, intralumenal vesicle formation and protein retention in Golgi apparatus, more
Afu5g13420	Chr5_A_fumigatus_Af293:3,529,175..3,531,137(-)	Has domain(s) with predicted ATP binding, protein kinase activity, protein tyrosine kinase activity and role in protein phosphorylation
Afu5g13440	Chr5_A_fumigatus_Af293:3,532,442..3,535,491(+)	protein of unknown function
Afu5g13450	Chr5_A_fumigatus_Af293:3,535,529..3,536,876(+)	Putative triosephosphate isomerase
Afu5g13460	Chr5_A_fumigatus_Af293:3,537,223..3,538,820(-)	Ortholog(s) have role in establishment of mitochondrion localization, mitochondrial outer membrane translocase complex assembly, mitochondrion distribution and mitochondrion morphogenesis, more
Afu5g13470	Chr5_A_fumigatus_Af293:3,538,964..3,539,883(+)	Ortholog(s) have role in cellular response to drug, nuclear-transcribed mRNA catabolic process, rRNA processing, ribosomal large subunit assembly and nucleolus, nucleoplasm, preribosome, large subunit precursor localization
Afu5g13480	Chr5_A_fumigatus_Af293:3,540,595..3,542,227(-)	Ortholog(s) have U1 snRNP, cytosol localization
Afu5g13490	Chr5_A_fumigatus_Af293:3,542,237..3,542,887(+)	Ortholog(s) have structural constituent of ribosome activity and mitochondrial large ribosomal subunit localization
Afu5g13500	Chr5_A_fumigatus_Af293:3,543,314..3,544,712(-)	Has domain(s) with predicted zinc ion binding activity
Afu5g13510	Chr5_A_fumigatus_Af293:3,544,752..3,546,764(-)	Ortholog(s) have Prp19 complex, cytosol, nuclear envelope, spliceosomal complex localization
Afu5g13520	Chr5_A_fumigatus_Af293:3,547,913..3,551,862(+)	Ortholog(s) have GTPase activity, role in mature ribosome assembly and cytosol localization
Afu5g13530	Chr5_A_fumigatus_Af293:3,551,872..3,553,268(-)	Ortholog(s) have role in exonucleolytic trimming to generate mature 3'-end of 5.8S rRNA from tricistronic rRNA transcript (SSU-rRNA, 5.8S rRNA and LSU-rRNA), more
Afu5g13540	Chr5_A_fumigatus_Af293:3,553,294..3,556,310(-)	Ortholog(s) have role in cellular response to biotic stimulus, filamentous growth of a population of unicellular organisms in response to biotic stimulus, hyphal growth, vacuole inheritance
Afu5g13550	Chr5_A_fumigatus_Af293:3,557,407..3,558,132(-)	Ortholog of Aspergillus fumigatus A1163 : AFUB_061250
Afu5g13560	Chr5_A_fumigatus_Af293:3,560,271..3,562,553(+)	Ortholog of A. nidulans FGSC A4 : AN6908, A. niger CBS 513.88 : An14g05050, A. oryzae RIB40 : AO090113000013, Aspergillus wentii : Aspwe1_0452655 and Aspergillus sydowii : Aspsy1_0589528
Afu5g13570	Chr5_A_fumigatus_Af293:3,564,070..3,565,005(+)	Ortholog of Aspergillus fumigatus A1163 : AFUB_061270 and Aspergillus niger ATCC 1015 : 41862-mRNA
Afu5g13580	Chr5_A_fumigatus_Af293:3,565,620..3,567,323(+)	Ortholog of A. oryzae RIB40 : AO090009000162, Aspergillus glaucus : Aspgl1_0157437, Neosartorya fischeri NRRL 181 : NFIA_073460 and Aspergillus clavatus NRRL 1 : ACLA_016570
Afu5g13590	Chr5_A_fumigatus_Af293:3,567,578..3,568,954(-)	protein of unknown function
Afu5g13600	Chr5_A_fumigatus_Af293:3,568,967..3,571,935(+)	Ortholog(s) have cytosol, nucleus localization
Afu5g13610	Chr5_A_fumigatus_Af293:3,571,974..3,572,617(-)	Ortholog of A. nidulans FGSC A4 : AN6912, A. niger CBS 513.88 : An14g05140, Aspergillus wentii : Aspwe1_0067652 and Aspergillus sydowii : Aspsy1_0075810
Afu5g13620	Chr5_A_fumigatus_Af293:3,572,784..3,575,128(+)	Ortholog(s) have ubiquitin-specific protease activity, role in protein deubiquitination and endoplasmic reticulum localization
Afu5g13630	Chr5_A_fumigatus_Af293:3,575,248..3,576,231(-)	Has domain(s) with predicted calcium ion binding activity
Afu5g13640	Chr5_A_fumigatus_Af293:3,576,727..3,578,641(+)	Has domain(s) with predicted role in cell redox homeostasis
Afu5g13650	Chr5_A_fumigatus_Af293:3,579,472..3,580,637(-)	Transcript up-regulated in conidia exposed to neutrophils
Afu5g13670	Chr5_A_fumigatus_Af293:3,590,279..3,591,480(-)	Ortholog of Aspergillus fumigatus A1163 : AFUB_061380
Afu5g13680	Chr5_A_fumigatus_Af293:3,591,557..3,592,103(-)	Ortholog of Aspergillus fumigatus A1163 : AFUB_061390
Afu5g13690	Chr5_A_fumigatus_Af293:3,595,959..3,596,339(+)	Ortholog of Neosartorya fischeri NRRL 181 : NFIA_073370, Aspergillus versicolor : Aspve1_0040392, Aspergillus fumigatus A1163 : AFUB_061410 and Aspergillus sydowii : Aspsy1_0043473
Afu5g13710	Chr5_A_fumigatus_Af293:3,600,517..3,603,725(-)	Putative carboxylesterase
Afu5g13715	Chr5_A_fumigatus_Af293:3,604,288..3,606,254(+)	Ortholog of A. nidulans FGSC A4 : AN3395, AN7406, A. niger CBS 513.88 : An02g14300, An14g03530, Aspergillus versicolor : Aspve1_0038904 and Aspergillus clavatus NRRL 1 : ACLA_076630
Afu5g13725	Chr5_A_fumigatus_Af293:3,608,573..3,611,821(+)	Ortholog of A. nidulans FGSC A4 : AN6413, AN6419, AN6946, AN5312, AN5664, AN8328, AN3241, AN10369, AN1930, AN2386, AN1738, AN11159, AN8984, AN8951, AN1540, AN8971, AN9387, AN9266, AN9306, AN1317, AN10886, AN7232, AN7774, AN12202
Afu5g13730	Chr5_A_fumigatus_Af293:3,612,257..3,613,798(-)	Ortholog of Aspergillus fumigatus A1163 : AFUB_061470
Afu5g13740	Chr5_A_fumigatus_Af293:3,615,778..3,616,943(+)	Has domain(s) with predicted catalytic activity
Afu5g13750	Chr5_A_fumigatus_Af293:3,617,376..3,618,452(+)	Has domain(s) with predicted calcium ion binding activity
Afu5g13760	Chr5_A_fumigatus_Af293:3,619,848..3,621,200(-)	protein of unknown function
Afu5g13770	Chr5_A_fumigatus_Af293:3,622,403..3,623,319(-)	Ortholog of Aspergillus fumigatus A1163 : AFUB_061510
Afu5g13780	Chr5_A_fumigatus_Af293:3,623,758..3,625,179(+)	Ortholog of A. nidulans FGSC A4 : AN5292, AN7640, A. fumigatus Af293 : Afu5g12490, A. niger CBS 513.88 : An14g06730 and A. oryzae RIB40 : AO090120000374, AO090020000661
Afu5g13790	Chr5_A_fumigatus_Af293:3,625,262..3,626,851(-)	Has domain(s) with predicted RNA polymerase II transcription factor activity, sequence-specific DNA binding, zinc ion binding activity, role in regulation of transcription, DNA-templated and nucleus localization
Afu5g13800	Chr5_A_fumigatus_Af293:3,627,171..3,627,956(-)	Has domain(s) with predicted FMN binding, oxidoreductase activity and role in oxidation-reduction process
Afu5g13810	Chr5_A_fumigatus_Af293:3,628,384..3,629,848(+)	Transulfuration enzyme family protein
Afu5g13820	Chr5_A_fumigatus_Af293:3,629,930..3,630,945(+)	Ortholog of Neosartorya fischeri NRRL 181 : NFIA_073110, Aspergillus fumigatus A1163 : AFUB_061560 and Aspergillus clavatus NRRL 1 : ACLA_016800
Afu5g13830	Chr5_A_fumigatus_Af293:3,631,406..3,633,004(+)	Ortholog(s) have galactosidase activity
Afu5g13840	Chr5_A_fumigatus_Af293:3,633,218..3,634,121(-)	Ortholog(s) have hydrolase activity, role in glycolytic process, nucleotide catabolic process, penicillin biosynthetic process, sterigmatocystin biosynthetic process and cytosol localization
Afu5g13850	Chr5_A_fumigatus_Af293:3,634,853..3,637,276(+)	Putative oligopeptide transporter
Afu5g13865	Chr5_A_fumigatus_Af293:3,637,668..3,640,022(-)	Ortholog(s) have ATP-dependent RNA helicase inhibitor activity, RNA binding activity
Afu5g13890	Chr5_A_fumigatus_Af293:3,640,458..3,641,207(-)	Ortholog(s) have role in asexual sporulation resulting in formation of a cellular spore, cellular response to pH, endosome organization, hyphal growth and intralumenal vesicle formation, more
Afu5g13920	Chr5_A_fumigatus_Af293:3,643,547..3,645,141(+)	Putative Hsp90 binding co-chaperone
Afu5g13930	Chr5_A_fumigatus_Af293:3,645,629..3,646,879(+)	Ortholog(s) have mRNA binding activity, role in cytoplasmic translation and cytoplasm, polysomal ribosome localization
Afu5g13940	Chr5_A_fumigatus_Af293:3,647,368..3,648,558(-)	Has domain(s) with predicted oxidoreductase activity and role in oxidation-reduction process
Afu5g13970	Chr5_A_fumigatus_Af293:3,650,128..3,656,167(+)	Ortholog(s) have role in conidiophore development, hyphal growth, sporocarp development involved in sexual reproduction, syncytium formation by plasma membrane fusion
Afu5g13990	Chr5_A_fumigatus_Af293:3,656,748..3,658,181(-)	Ortholog of A. nidulans FGSC A4 : AN2703, A. niger CBS 513.88 : An14g05420, A. oryzae RIB40 : AO090113000104, Aspergillus wentii : Aspwe1_0453318 and Aspergillus sydowii : Aspsy1_0155176
Afu5g13995	Chr5_A_fumigatus_Af293:3,658,304..3,659,254(-)	Has domain(s) with predicted hydrolase activity, hydrolyzing O-glycosyl compounds activity and role in carbohydrate metabolic process
Afu5g14000	Chr5_A_fumigatus_Af293:3,659,835..3,661,209(-)	Has domain(s) with predicted oxidoreductase activity and role in metabolic process
Afu5g14010	Chr5_A_fumigatus_Af293:3,661,317..3,663,206(+)	Ortholog(s) have role in snoRNA metabolic process and cytoplasm, nucleus localization
Afu5g14020	Chr5_A_fumigatus_Af293:3,663,219..3,665,761(-)	Ortholog(s) have nuclear periphery localization
Afu5g14030	Chr5_A_fumigatus_Af293:3,666,645..3,668,270(+)	Ortholog of A. nidulans FGSC A4 : AN2690, A. niger CBS 513.88 : An14g05490, A. oryzae RIB40 : AO090113000118, Aspergillus wentii : Aspwe1_0453526 and Aspergillus sydowii : Aspsy1_0155771
Afu5g14040	Chr5_A_fumigatus_Af293:3,668,387..3,672,523(-)	Ortholog(s) have SUMO-specific protease activity and role in protein desumoylation
Afu5g14050	Chr5_A_fumigatus_Af293:3,672,792..3,675,072(-)	Has domain(s) with predicted catalytic activity and membrane localization
Afu5g14060	Chr5_A_fumigatus_Af293:3,676,056..3,677,836(+)	Putative Rho-type GTPase
Afu5g14070	Chr5_A_fumigatus_Af293:3,678,014..3,678,515(-)	Has domain(s) with predicted role in mitotic spindle organization in nucleus and DASH complex, mitotic spindle localization
Afu5g14080	Chr5_A_fumigatus_Af293:3,679,540..3,680,694(-)	Ortholog of A. nidulans FGSC A4 : AN2640, A. niger CBS 513.88 : An12g05640, Neosartorya fischeri NRRL 181 : NFIA_072900 and Aspergillus versicolor : Aspve1_0040725
Afu5g14090	Chr5_A_fumigatus_Af293:3,682,264..3,685,263(-)	Has domain(s) with predicted catalytic activity, hydrolase activity, hydrolyzing O-glycosyl compounds activity and role in carbohydrate metabolic process

Afu5g14100	Chr5_A_fumigatus_Af293:3,686,207..3,687,150(-)	Ortholog of A. niger CBS 513.88 : An02g01070, Aspergillus brasiliensis : Aspbr1_0072526, Aspergillus flavus NRRL 3357 : AFL2T_11035 and Neosartorya fischeri NRRL 181 : NFIA_072860
Afu5g14110	Chr5_A_fumigatus_Af293:3,687,288..3,687,656(-)	Ortholog of Aspergillus fumigatus A1163 : AFUB_061800
Afu5g14120	Chr5_A_fumigatus_Af293:3,687,913..3,688,824(-)	Ortholog(s) have cytosol, nucleus localization
Afu5g14130	Chr5_A_fumigatus_Af293:3,689,498..3,690,581(+)	Ortholog of A. nidulans FGSC A4 : AN9531, AN1600, AN9354, AN4608 and A. fumigatus Af293 : Afu4g03520, Afu7g06920
Afu5g14140	Chr5_A_fumigatus_Af293:3,690,998..3,693,368(+)	Has domain(s) with predicted DNA binding, RNA polymerase II transcription factor activity, sequence-specific DNA binding, zinc ion binding activity and role in regulation of transcription, DNA-templated, transcription, DNA-templated
Afu5g14150	Chr5_A_fumigatus_Af293:3,694,494..3,696,099(+)	Has domain(s) with predicted triglyceride lipase activity and role in lipid catabolic process
Afu5g14160	Chr5_A_fumigatus_Af293:3,697,530..3,698,134(-)	Ortholog of Aspergillus fumigatus A1163 : AFUB_061850
Afu5g14170	Chr5_A_fumigatus_Af293:3,699,067..3,699,625(-)	Ortholog of Aspergillus fumigatus A1163 : AFUB_061860
Afu5g14180	Chr5_A_fumigatus_Af293:3,699,978..3,701,185(+)	Ortholog of A. nidulans FGSC A4 : AN7186, A. niger CBS 513.88 : An14g05580, A. oryzae RIB40 : AO090001000217, Aspergillus wentii : Aspwe1_0179974 and Aspergillus sydowii : Aspsy1_0026595
Afu5g14190	Chr5_A_fumigatus_Af293:3,701,409..3,703,309(+)	Beta gluconase
Afu5g14200	Chr5_A_fumigatus_Af293:3,703,586..3,705,688(+)	Ortholog of A. nidulans FGSC A4 : AN0196, A. fumigatus Af293 : Afu6g13810, Afu7g05110, Afu8g01720, A. niger CBS 513.88 : An13g02610, An15g02090, An04g09640 and A. oryzae RIB40 : AO090003001444
Afu5g14210	Chr5_A_fumigatus_Af293:3,705,868..3,706,130(-)	Glucose-repressible gene
Afu5g14220	Chr5_A_fumigatus_Af293:3,706,433..3,707,309(-)	Ortholog of Aspergillus fumigatus A1163 : AFUB_061920
Afu5g14230	Chr5_A_fumigatus_Af293:3,708,234..3,709,929(-)	Has domain(s) with predicted RNA polymerase II transcription factor activity, sequence-specific DNA binding, transcription factor activity, sequence-specific DNA binding, zinc ion binding activity
Afu5g14240	Chr5_A_fumigatus_Af293:3,712,963..3,713,643(+)	Ortholog of A. nidulans FGSC A4 : AN6760, AN8527, A. fumigatus Af293 : Afu4g01400, Afu5g02670, A. niger CBS 513.88 : An07g09050, An08g09630 and Aspergillus wentii : Aspwe1_0069899, Aspwe1_0185315, Aspwe1_0186212
Afu5g14250	Chr5_A_fumigatus_Af293:3,714,031..3,714,984(+)	Ortholog of A. nidulans FGSC A4 : AN7782, A. niger CBS 513.88 : An14g05590, A. oryzae RIB40 : AO090001000199, Aspergillus wentii : Aspwe1_0028859 and Aspergillus sydowii : Aspsy1_0046329
Afu5g14260	Chr5_A_fumigatus_Af293:3,715,059..3,715,517(+)	Ortholog(s) have electron carrier activity, role in ergosterol biosynthetic process and endoplasmic reticulum membrane localization
Afu5g14270	Chr5_A_fumigatus_Af293:3,715,548..3,717,820(-)	Has domain(s) with predicted catalytic activity and role in metabolic process
Afu5g14280	Chr5_A_fumigatus_Af293:3,718,196..3,719,894(-)	Ortholog of Aspergillus fumigatus A1163 : AFUB_061990
Afu5g14290	Chr5_A_fumigatus_Af293:3,720,488..3,723,757(-)	Has domain(s) with predicted DNA binding, RNA polymerase II transcription factor activity, sequence-specific DNA binding, zinc ion binding activity and role in regulation of transcription, DNA-templated, transcription, DNA-templated
Afu5g14300	Chr5_A_fumigatus_Af293:3,728,017..3,730,042(+)	Putative trehalose-6-phosphate synthase
Afu5g14310	Chr5_A_fumigatus_Af293:3,730,662..3,731,858(+)	Has domain(s) with predicted oxidoreductase activity and role in metabolic process
Afu5g14315	Chr5_A_fumigatus_Af293:3,731,908..3,733,116(-)	Ortholog of A. oryzae RIB40 : AO090701000372, Neosartorya fischeri NRRL 181 : NFIA_072570, Aspergillus wentii : Aspwe1_0042955, Aspwe1_0063639 and Aspergillus versicolor : Aspve1_0035102, Aspve1_0087828
Afu5g14320	Chr5_A_fumigatus_Af293:3,733,224..3,735,879(-)	Ortholog of A. nidulans FGSC A4 : AN2683, AN5069, A. fumigatus Af293 : Afu5g02860, A. niger CBS 513.88 : An09g04830, An14g05730 and A. oryzae RIB40 : AO090102000482, AO090001000197
Afu5g14330	Chr5_A_fumigatus_Af293:3,736,232..3,737,853(+)	12-oxophytodienoate reductase
Afu5g14340	Chr5_A_fumigatus_Af293:3,737,907..3,738,880(-)	Has domain(s) with predicted oxidoreductase activity and role in metabolic process
Afu5g14345	Chr5_A_fumigatus_Af293:3,739,414..3,739,623(-)	Ortholog of Aspergillus fumigatus A1163 : AFUB_062070
Afu5g14350	Chr5_A_fumigatus_Af293:3,739,719..3,741,228(+)	C-24(28) sterol reductase
Afu5g14360	Chr5_A_fumigatus_Af293:3,741,300..3,743,416(-)	Has domain(s) with predicted DNA binding, zinc ion binding activity, role in transcription, DNA-templated and nucleus localization
Afu5g14380	Chr5_A_fumigatus_Af293:3,744,313..3,746,835(+)	Alpha-glucuronidase
Afu5g14390	Chr5_A_fumigatus_Af293:3,747,371..3,749,191(-)	Putative C6 transcription factor
Afu5g14410	Chr5_A_fumigatus_Af293:3,749,485..3,751,272(+)	Putative cysteine dioxygenase
Afu5g14420	Chr5_A_fumigatus_Af293:3,751,275..3,753,764(-)	Ortholog of A. nidulans FGSC A4 : AN9361, A. niger CBS 513.88 : An05g01800, A. oryzae RIB40 : AO090103000493, Aspergillus wentii : Aspwe1_0041204 and Aspergillus sydowii : Aspsy1_0051612
Afu5g14490	Chr5_A_fumigatus_Af293:3,760,906..3,762,694(+)	Has domain(s) with predicted role in transmembrane transport and integral component of membrane localization
Afu5g14500	Chr5_A_fumigatus_Af293:3,762,799..3,765,108(-)	Has domain(s) with predicted role in transmembrane transport and integral component of membrane localization
Afu5g14510	Chr5_A_fumigatus_Af293:3,765,129..3,766,445(-)	Ortholog of A. niger CBS 513.88 : An06g02470, Aspergillus brasiliensis : Aspbr1_0060254, Neosartorya fischeri NRRL 181 : NFIA_072450 and Aspergillus fumigatus A1163 : AFUB_062190
Afu5g14520	Chr5_A_fumigatus_Af293:3,767,027..3,768,238(+)	Ortholog of Neosartorya fischeri NRRL 181 : NFIA_072440, Aspergillus fumigatus A1163 : AFUB_062200, Aspergillus clavatus NRRL 1 : ACLA_017360 and Aspergillus zonatus : Aspzo1_0136732
Afu5g14530	Chr5_A_fumigatus_Af293:3,768,297..3,770,530(-)	Ortholog(s) have hydrolase activity, hydrolyzing O-glycosyl compounds activity and role in cellulose catabolic process, positive regulation of cellulose catabolic process, regulation of glycogen metabolic process
Afu5g14540	Chr5_A_fumigatus_Af293:3,770,727..3,772,681(-)	Has domain(s) with predicted substrate-specific transmembrane transporter activity, transmembrane transporter activity, role in transmembrane transport and integral component of membrane, membrane localization
Afu5g14550	Chr5_A_fumigatus_Af293:3,773,063..3,776,828(+)	Ortholog(s) have role in lactose metabolic process
Afu5g14560	Chr5_A_fumigatus_Af293:3,777,106..3,778,726(+)	Has domain(s) with predicted catalytic activity, hydrolase activity, hydrolyzing O-glycosyl compounds activity and role in carbohydrate metabolic process
Afu5g14570	Chr5_A_fumigatus_Af293:3,778,950..3,780,479(-)	Ortholog of A. nidulans FGSC A4 : AN3322, Neosartorya fischeri NRRL 181 : NFIA_072390 and Aspergillus fumigatus A1163 : AFUB_062250
Afu5g14582	Chr5_A_fumigatus_Af293:3,781,952..3,785,288(-)	Has domain(s) with predicted catalytic activity and role in nucleoside metabolic process
Afu5g14600	Chr5_A_fumigatus_Af293:3,786,987..3,787,774(+)	Ortholog of A. nidulans FGSC A4 : AN8144, AN1651, A. fumigatus Af293 : Afu6g00460, Afu6g13950, A. niger CBS 513.88 : An11g02490, An01g11420 and A. oryzae RIB40 : AO090001000384, AO090011000740
Afu5g14610	Chr5_A_fumigatus_Af293:3,788,121..3,789,763(-)	Putative carboxypeptidase Y
Afu5g14620	Chr5_A_fumigatus_Af293:3,790,328..3,790,915(-)	Ortholog of Aspergillus fumigatus A1163 : AFUB_062290
Afu5g14630	Chr5_A_fumigatus_Af293:3,790,987..3,791,391(+)	Ortholog of Aspergillus fumigatus A1163 : AFUB_062300
Afu5g14640	Chr5_A_fumigatus_Af293:3,791,791..3,792,708(+)	Has domain(s) with predicted hydrolase activity, hydrolase activity, acting on acid halide bonds, in C-halide compounds activity and role in metabolic process
Afu5g14650	Chr5_A_fumigatus_Af293:3,794,320..3,794,669(+)	Putative RING finger protein
Afu5g14660	Chr5_A_fumigatus_Af293:3,794,749..3,797,326(-)	Ortholog of A. nidulans FGSC A4 : AN3345, AN3347, A. fumigatus Af293 : Afu5g00710, A. niger CBS 513.88 : An16g06090, An12g10000/gabA and A. oryzae RIB40 : AO090166000026, AO090005000509, AO090005001081
Afu5g14670	Chr5_A_fumigatus_Af293:3,798,029..3,799,324(+)	Ortholog of A. niger CBS 513.88 : An12g09990 and Aspergillus fumigatus A1163 : AFUB_062340
Afu5g14680	Chr5_A_fumigatus_Af293:3,799,759..3,800,442(+)	Protein with Yap1-dependent induction in response to hydrogen peroxide
Afu5g14690	Chr5_A_fumigatus_Af293:3,800,550..3,801,486(-)	Has domain(s) with predicted ATP adenylyltransferase activity
Afu5g14700	Chr5_A_fumigatus_Af293:3,801,990..3,802,183(-)	Ortholog of Aspergillus fumigatus A1163 : AFUB_062370
Afu5g14710	Chr5_A_fumigatus_Af293:3,802,659..3,804,069(+)	Ortholog of Neosartorya fischeri NRRL 181 : NFIA_072270, Aspergillus versicolor : Aspve1_0129744, Aspergillus fumigatus A1163 : AFUB_062380 and Aspergillus clavatus NRRL 1 : ACLA_017450
Afu5g14720	Chr5_A_fumigatus_Af293:3,804,392..3,805,903(-)	Ortholog of A. nidulans FGSC A4 : AN7942, AN7872, AN5510, AN3420, AN2700, AN9050, AN8973, AN11222, AN10911, AN1749 and A. fumigatus Af293 : Afu1g12450, Afu2g00880, Afu2g17800, Afu2g17880, Afu7g06590
Afu5g14730	Chr5_A_fumigatus_Af293:3,806,706..3,808,068(+)	Has domain(s) with predicted oxidoreductase activity and role in oxidation-reduction process
Afu5g14740	Chr5_A_fumigatus_Af293:3,810,067..3,811,228(+)	L-fucose-specific lectin
Afu5g14750	Chr5_A_fumigatus_Af293:3,811,726..3,812,598(+)	Ortholog of A. nidulans FGSC A4 : AN0198, A. niger CBS 513.88 : An14g07220, Aspergillus wentii : Aspwe1_0043650, Aspwe1_0044769 and Aspergillus sydowii : Aspsy1_0040680
Afu5g14760	Chr5_A_fumigatus_Af293:3,812,828..3,813,315(+)	Ortholog of A. niger CBS 513.88 : An05g01370, A. oryzae RIB40 : AO090001000123, Neosartorya fischeri NRRL 181 : NFIA_072220 and Aspergillus wentii : Aspwe1_0724935
Afu5g14770	Chr5_A_fumigatus_Af293:3,814,249..3,815,513(-)	Has domain(s) with predicted oxidoreductase activity, pyrroline-5-carboxylate reductase activity and role in oxidation-reduction process, proline biosynthetic process
Afu5g14780	Chr5_A_fumigatus_Af293:3,818,808..3,821,303(+)	Putative trehalase phosphorylase with a predicted role in the glucose-1-phosphate pathway
Afu5g14790	Chr5_A_fumigatus_Af293:3,822,466..3,824,886(+)	Putative phosphoenol pyruvate synthase
Afu5g14800	Chr5_A_fumigatus_Af293:3,825,104..3,826,608(-)	Lactate dehydrogenase
Afu5g14810	Chr5_A_fumigatus_Af293:3,828,015..3,830,041(+)	Putative pyruvate decarboxylase

Afu5g14820	Chr5_A_fumigatus_Af293:3,830,428..3,832,037(-)	Has domain(s) with predicted ATP binding, nucleoside-triphosphatase activity, nucleotide binding activity
Afu5g14830	Chr5_A_fumigatus_Af293:3,833,301..3,833,733(-)	Ortholog of A. nidulans FGSC A4 : AN8989, A. oryzae RIB40 : AO090001000186, Neosartorya fischeri NRRL 181 : NFIA_041290 and Aspergillus wentii : Aspwe1_0039524, Aspwe1_0045369
Afu5g14840	Chr5_A_fumigatus_Af293:3,834,455..3,835,135(-)	Ortholog of A. nidulans FGSC A4 : AN4127, A. fumigatus Af293 : Afu5g14680, A. niger CBS 513.88 : An15g05400, Neosartorya fischeri NRRL 181 : NFIA_072290 and Aspergillus wentii : Aspwe1_0041184
Afu5g14845	Chr5_A_fumigatus_Af293:3,836,427..3,837,563(+)	Has domain(s) with predicted zinc ion binding activity
Afu5g14850	Chr5_A_fumigatus_Af293:3,838,802..3,840,474(-)	protein of unknown function
Afu5g14860	Chr5_A_fumigatus_Af293:3,841,417..3,842,238(-)	Has domain(s) with predicted electron carrier activity, heme binding, iron ion binding, oxidoreductase activity, acting on paired donors, with incorporation or reduction of molecular oxygen activity and role in oxidation-reduction process
Afu5g14865	Chr5_A_fumigatus_Af293:3,842,373..3,845,049(+)	protein of unknown function
Afu5g14870	Chr5_A_fumigatus_Af293:3,845,841..3,847,633(-)	Putative protein serine/threonine kinase
Afu5g14880	Chr5_A_fumigatus_Af293:3,847,948..3,849,477(-)	Ortholog(s) have mitochondrion localization
Afu5g14890	Chr5_A_fumigatus_Af293:3,849,926..3,851,084(-)	Ortholog of A. nidulans FGSC A4 : AN4143, AN12170, A. fumigatus Af293 : Afu1g00760, Aspergillus wentii : Aspwe1_0177868, Aspwe1_0403846 and Aspergillus niger ATCC 1015 : 41666-mRNA
Afu5g14900	Chr5_A_fumigatus_Af293:3,851,274..3,852,983(+)	Ortholog of Aspergillus fumigatus A1163 : AFUB_079010
Afu5g14910	Chr5_A_fumigatus_Af293:3,853,365..3,853,944(-)	Ortholog of Aspergillus fumigatus A1163 : AFUB_079020
Afu5g14920	Chr5_A_fumigatus_Af293:3,857,350..3,859,552(+)	protein of unknown function
Afu5g14930	Chr5_A_fumigatus_Af293:3,862,407..3,865,284(-)	protein of unknown function
Afu5g14940	Chr5_A_fumigatus_Af293:3,867,797..3,869,542(+)	Putative cell surface metalloreductase
Afu5g14950	Chr5_A_fumigatus_Af293:3,870,635..3,872,465(+)	protein of unknown function
Afu5g14960	Chr5_A_fumigatus_Af293:3,873,055..3,873,408(-)	Ortholog of Aspergillus fumigatus A1163 : AFUB_079070
Afu5g14970	Chr5_A_fumigatus_Af293:3,873,578..3,874,273(-)	Ortholog of A. oryzae RIB40 : AO090010000299 and Aspergillus fumigatus A1163 : AFUB_079080, AFUB_096660
Afu5g14980	Chr5_A_fumigatus_Af293:3,874,539..3,877,890(+)	Conserved protein of unknown function
Afu5g14990	Chr5_A_fumigatus_Af293:3,878,797..3,880,632(-)	protein of unknown function
Afu5g15000	Chr5_A_fumigatus_Af293:3,880,881..3,882,138(-)	Putative arsenate reductase
Afu5g15010	Chr5_A_fumigatus_Af293:3,882,249..3,883,746(+)	Putative arsenite efflux transporter
Afu5g15020	Chr5_A_fumigatus_Af293:3,883,929..3,886,741(-)	Arsenic methyltransferase
Afu5g15030	Chr5_A_fumigatus_Af293:3,886,790..3,888,430(-)	Putative Arsenic resistance protein
Afu5g15040	Chr5_A_fumigatus_Af293:3,891,893..3,895,481(-)	Ortholog of A. nidulans FGSC A4 : AN3546, A. fumigatus Af293 : Afu1g16170, Afu4g14400, Afu6g09300, A. niger CBS 513.88 : An08g11160, An04g08000 and A. oryzae RIB40 : AO090166000001
Afu5g15050	Chr5_A_fumigatus_Af293:3,895,482..3,896,523(-)	Has domain(s) with predicted electron carrier activity, heme binding, iron ion binding, oxidoreductase activity, acting on paired donors, with incorporation or reduction of molecular oxygen activity and role in oxidation-reduction process
Afu5g15060	Chr5_A_fumigatus_Af293:3,896,707..3,900,558(+)	Ortholog of A. nidulans FGSC A4 : AN1594, AN9314, A. niger CBS 513.88 : An18g02710, A. oryzae RIB40 : AO090023000073, Neosartorya fischeri NRRL 181 : NFIA_009790 and Aspergillus wentii : Aspwe1_0054582
Afu5g15070	Chr5_A_fumigatus_Af293:3,901,027..3,902,701(-)	Has domain(s) with predicted antioxidant activity, oxidoreductase activity, peroxiredoxin activity and role in oxidation-reduction process
Afu5g15080	Chr5_A_fumigatus_Af293:3,906,826..3,907,128(-)	Has domain(s) with predicted ATP binding, protein kinase activity and role in protein phosphorylation
Afu5g15140	Chr5_A_fumigatus_Af293:3,934,049..3,934,255(-)	protein of unknown function
Afu5g15150	Chr5_A_fumigatus_Af293:3,942,655..3,944,487(-)	protein of unknown function

Gene ID	Genomic Location (Gene)	Product Description
Afu4g06160	Chr4_A_fumigatus_Af293:1,580,191..1,583,864(+)	Branched-chain-amino-acid transaminase
Afu4g06170	Chr4_A_fumigatus_Af293:1,588,352..1,590,771(+)	Has domain(s) with predicted sequence-specific DNA binding, transcription factor activity, sequence-specific DNA binding activ
Afu4g06180	Chr4_A_fumigatus_Af293:1,591,035..1,595,980(-)	Ortholog(s) have protein serine/threonine kinase inhibitor activity
Afu4g06190	Chr4_A_fumigatus_Af293:1,596,221..1,598,467(-)	Putative fungal specific transcription factor
Afu4g06200	Chr4_A_fumigatus_Af293:1,599,168..1,601,178(-)	Ortholog of A. nidulans FGSC A4 : AN6683, AN8537, AN5260 and A. niger CBS 513.88 : An12g04370, An11g06510, An14g0126
Afu4g06210	Chr4_A_fumigatus_Af293:1,601,764..1,603,146(+)	Ortholog of A. nidulans FGSC A4 : AN10542, A. niger CBS 513.88 : An04g00510, A. oryzae RIB40 : AO090023000969, Aspergillus
Afu4g06220	Chr4_A_fumigatus_Af293:1,603,168..1,603,958(+)	Ortholog of A. nidulans FGSC A4 : AN10547, A. oryzae RIB40 : AO090023000968, Neosartorya fischeri NRRL 181 : NFIA_109920
Afu4g06230	Chr4_A_fumigatus_Af293:1,604,141..1,607,360(-)	Ortholog(s) have role in positive regulation of (R)-carnitine transmembrane transport, positive regulation of polyamine transme
Afu4g06240	Chr4_A_fumigatus_Af293:1,609,280..1,609,811(+)	Has domain(s) with predicted heme binding activity
Afu4g06250	Chr4_A_fumigatus_Af293:1,610,044..1,612,266(-)	Ortholog(s) have role in cellular response to drug and nucleolus localization
Afu4g06260	Chr4_A_fumigatus_Af293:1,612,405..1,614,216(+)	Ortholog(s) have polyubiquitin binding activity and role in double-strand break repair via single-strand annealing, removal of nc
Afu4g06275	Chr4_A_fumigatus_Af293:1,614,220..1,617,160(+)	Ortholog of A. nidulans FGSC A4 : AN0227, A. fumigatus Af293 : Afu3g12020, Afu5g10910, A. niger CBS 513.88 : An14g03700, A
Afu4g06290	Chr4_A_fumigatus_Af293:1,617,227..1,619,928(-)	Ortholog(s) have sphingosine N-acyltransferase activity and role in filamentous growth of a population of unicellular organisms,
Afu4g06300	Chr4_A_fumigatus_Af293:1,619,945..1,621,703(-)	Ortholog of A. nidulans FGSC A4 : AN4333, A. oryzae RIB40 : AO090023000961, Neosartorya fischeri NRRL 181 : NFIA_109850, A
Afu4g06310	Chr4_A_fumigatus_Af293:1,622,288..1,622,838(-)	Ortholog of Aspergillus fumigatus A1163 : AFUB_063430
Afu4g06320	Chr4_A_fumigatus_Af293:1,624,724..1,625,106(+)	Ortholog of A. nidulans FGSC A4 : AN4340, A. niger CBS 513.88 : An04g00650, Neosartorya fischeri NRRL 181 : NFIA_109830 an
Afu4g06330	Chr4_A_fumigatus_Af293:1,625,616..1,626,767(-)	Ortholog of A. nidulans FGSC A4 : AN4341, A. niger CBS 513.88 : An04g00660, A. oryzae RIB40 : AO090023000957, Aspergillus
Afu4g06340	Chr4_A_fumigatus_Af293:1,627,154..1,627,946(+)	Ortholog(s) have endoplasmic reticulum localization
Afu4g06350	Chr4_A_fumigatus_Af293:1,628,898..1,630,750(-)	Has domain(s) with predicted nucleic acid binding, nucleotide binding activity
Afu4g06360	Chr4_A_fumigatus_Af293:1,630,810..1,632,062(+)	Has domain(s) with predicted RNA binding activity, role in mRNA processing and cytoplasm, nucleus localization
Afu4g06370	Chr4_A_fumigatus_Af293:1,632,129..1,634,165(-)	Ortholog of A. niger CBS 513.88 : An04g00710, Neosartorya fischeri NRRL 181 : NFIA_109780, Aspergillus wentii : Aspwe1_002
Afu4g06380	Chr4_A_fumigatus_Af293:1,634,195..1,636,139(-)	Putative sterol carrier protein
Afu4g06390	Chr4_A_fumigatus_Af293:1,638,303..1,639,644(+)	Ortholog(s) have mitochondrion localization
Afu4g06400	Chr4_A_fumigatus_Af293:1,640,020..1,642,754(+)	Ortholog(s) have role in cellular response to lithium ion, cellular response to neutral pH, entry into host and filamentous growth
Afu4g06410	Chr4_A_fumigatus_Af293:1,642,832..1,643,843(-)	Ortholog of Neosartorya fischeri NRRL 181 : NFIA_109740, Aspergillus wentii : Aspwe1_0023328, Aspergillus fumigatus A1163 :
Afu4g06420	Chr4_A_fumigatus_Af293:1,643,911..1,646,582(-)	Putative fungal specific transcription factor
Afu4g06430	Chr4_A_fumigatus_Af293:1,647,247..1,647,828(-)	Ortholog of Aspergillus fumigatus A1163 : AFUB_063549
Afu4g06460	Chr4_A_fumigatus_Af293:1,650,012..1,653,136(-)	Has domain(s) with predicted 3-dehydroquinate dehydratase activity, catalytic activity
Afu4g06480	Chr4_A_fumigatus_Af293:1,654,167..1,658,353(-)	Has domain(s) with predicted role in intracellular signal transduction
Afu4g06490	Chr4_A_fumigatus_Af293:1,661,540..1,664,377(-)	Ortholog(s) have role in meiotic mismatch repair, reciprocal meiotic recombination and MutLgamma complex, nucleus localiza
Afu4g06500	Chr4_A_fumigatus_Af293:1,665,914..1,667,157(-)	Ortholog of A. nidulans FGSC A4 : AN4362, A. oryzae RIB40 : AO090023000936, Neosartorya fischeri NRRL 181 : NFIA_003290,
Afu4g06510	Chr4_A_fumigatus_Af293:1,667,731..1,671,333(-)	Ortholog of A. nidulans FGSC A4 : AN1972, AN4371, A. niger CBS 513.88 : An04g05970, An08g04550, A. oryzae RIB40 : AO0900
Afu4g06520	Chr4_A_fumigatus_Af293:1,671,673..1,673,936(+)	Ortholog of A. nidulans FGSC A4 : AN4364, A. niger CBS 513.88 : An04g00860, A. oryzae RIB40 : AO090023000934, Aspergillus
Afu4g06530	Chr4_A_fumigatus_Af293:1,676,511..1,679,413(-)	bZip transcription factor, putative
Afu4g06540	Chr4_A_fumigatus_Af293:1,680,790..1,682,202(+)	Ortholog(s) have structural molecule activity
Afu4g06550	Chr4_A_fumigatus_Af293:1,682,651..1,684,353(-)	Has domain(s) with predicted zinc ion binding activity
Afu4g06560	Chr4_A_fumigatus_Af293:1,684,493..1,686,070(+)	Ortholog of Aspergillus fumigatus A1163 : AFUB_063640 and Aspergillus terreus NIH2624 : ATET_05579

Afu4g06570	Chr4_A_fumigatus_Af293:1,686,920..1,690,724(-)	Ortholog(s) have hyphal tip localization
Afu4g06580	Chr4_A_fumigatus_Af293:1,691,358..1,691,597(+)	Ortholog of Aspergillus fumigatus A1163 : AFUB_063660
Afu4g06590	Chr4_A_fumigatus_Af293:1,695,590..1,699,886(+)	Ortholog of Neosartorya fischeri NRRL 181 : NFIA_109600 and Aspergillus fumigatus A1163 : AFUB_063670
Afu4g06600	Chr4_A_fumigatus_Af293:1,700,116..1,703,381(-)	Has domain(s) with predicted RNA polymerase II transcription cofactor activity, role in regulation of transcription from RNA pol
Afu4g06610	Chr4_A_fumigatus_Af293:1,703,777..1,707,458(-)	Ortholog of A. nidulans FGSC A4 : AN2913, AN4375, A. fumigatus Af293 : Afu3g07740, A. niger CBS 513.88 : An04g00930 and A.
Afu4g06620	Chr4_A_fumigatus_Af293:1,711,767..1,714,021(+)	Glutamate/Leucine/Phenylalanine/Valine dehydrogenase
Afu4g06630	Chr4_A_fumigatus_Af293:1,714,079..1,717,387(-)	Has domain(s) with predicted role in transmembrane transport and integral component of membrane localization
Afu4g06640	Chr4_A_fumigatus_Af293:1,719,069..1,721,809(+)	Has domain(s) with predicted hydrolase activity
Afu4g06660	Chr4_A_fumigatus_Af293:1,725,435..1,726,346(-)	Ortholog of Aspergillus fumigatus A1163 : AFUB_063740
Afu4g06670	Chr4_A_fumigatus_Af293:1,726,811..1,728,795(-)	Allergen Asp f 7
Afu4g06690	Chr4_A_fumigatus_Af293:1,730,762..1,734,332(-)	Ortholog(s) have CDP reductase activity, nucleoside diphosphate kinase activity, nucleotide binding activity
Afu4g06700	Chr4_A_fumigatus_Af293:1,734,334..1,735,654(-)	Ortholog of A. nidulans FGSC A4 : AN1338, AN9450, AN4381, A. fumigatus Af293 : Afu1g03040, Afu1g09510 and A. niger CBS 5
Afu4g06710	Chr4_A_fumigatus_Af293:1,735,766..1,737,047(-)	Ortholog(s) have ATPase activity and cytosol, nucleus localization
Afu4g06730	Chr4_A_fumigatus_Af293:1,737,114..1,739,076(-)	Ortholog of A. nidulans FGSC A4 : AN4383, A. niger CBS 513.88 : An04g01110, A. oryzae RIB40 : AO090023000913, Aspergillus
Afu4g06740	Chr4_A_fumigatus_Af293:1,739,546..1,741,125(+)	Ortholog(s) have cytosol, mitochondrion localization
Afu4g06750	Chr4_A_fumigatus_Af293:1,741,249..1,746,859(+)	Ortholog(s) have role in actomyosin contractile ring assembly, ascospore formation, sporocarp development involved in sexual
Afu4g06760	Chr4_A_fumigatus_Af293:1,746,944..1,749,433(+)	Ortholog(s) have role in lipid homeostasis, mitochondrion organization and integral component of mitochondrial membrane, r
Afu4g06770	Chr4_A_fumigatus_Af293:1,749,552..1,751,095(-)	Ortholog(s) have 2 iron, 2 sulfur cluster binding, ferrous iron binding, iron-sulfur transferase activity
Afu4g06780	Chr4_A_fumigatus_Af293:1,752,595..1,754,935(-)	Ortholog(s) have NAD transporter activity, pyruvate secondary active transmembrane transporter activity, role in NAD transmer
Afu4g06790	Chr4_A_fumigatus_Af293:1,755,390..1,756,027(+)	Ubiquinol-cytochrome c reductase complex 14 kDa protein with a predicted role in oxidative phosphorylation
Afu4g06800	Chr4_A_fumigatus_Af293:1,756,157..1,758,180(-)	Ortholog(s) have DNA-3-methyladenine glycosylase activity, damaged DNA binding activity, role in DNA dealkylation involved in
Afu4g06810	Chr4_A_fumigatus_Af293:1,761,636..1,761,791(-)	Ortholog of Aspergillus fumigatus A1163 : AFUB_063880
Afu4g06820	Chr4_A_fumigatus_Af293:1,762,193..1,763,534(+)	Putative glycophosphatidylinositol (GPI)-anchored cell wall protein with similarity to S. cerevisiae Ecm33p
Afu4g06830	Chr4_A_fumigatus_Af293:1,764,215..1,765,322(-)	Ortholog(s) have SUMO transferase activity, role in hyphal growth, mitotic spindle elongation, protein sumoylation and conden
Afu4g06840	Chr4_A_fumigatus_Af293:1,765,448..1,768,207(-)	Has domain(s) with predicted nucleic acid binding, nucleotide binding activity
Afu4g06850	Chr4_A_fumigatus_Af293:1,768,319..1,770,087(+)	Ortholog(s) have cytosol, glyoxysome localization
Afu4g06860	Chr4_A_fumigatus_Af293:1,770,090..1,770,590(-)	Ortholog of Aspergillus fumigatus A1163 : AFUB_063930
Afu4g06870	Chr4_A_fumigatus_Af293:1,771,306..1,774,301(-)	Ortholog of A. nidulans FGSC A4 : AN4395, A. niger CBS 513.88 : An04g01260, A. oryzae RIB40 : AO090023000901, Aspergillus
Afu4g06880	Chr4_A_fumigatus_Af293:1,774,383..1,776,973(-)	protein of unknown function
Afu4g06890	Chr4_A_fumigatus_Af293:1,780,884..1,785,331(-)	14-alpha sterol demethylase
Afu4g06900	Chr4_A_fumigatus_Af293:1,787,004..1,789,825(+)	Ortholog(s) have asparagine synthase (glutamine-hydrolyzing) activity, role in asparagine biosynthetic process and cytosol, nucl
Afu4g06910	Chr4_A_fumigatus_Af293:1,789,863..1,791,391(+)	Putative outer mitochondrial membrane protein porin
Afu4g06920	Chr4_A_fumigatus_Af293:1,791,595..1,793,040(-)	Ortholog of A. nidulans FGSC A4 : AN4403, Aspergillus wentii : Aspwe1_0167198, Aspergillus sydowii : Aspsy1_0162965 and As
Afu4g06930	Chr4_A_fumigatus_Af293:1,793,635..1,795,535(+)	Ortholog(s) have cytosol localization
Afu4g06940	Chr4_A_fumigatus_Af293:1,795,602..1,797,234(+)	Ortholog(s) have sphingolipid delta-4 desaturase activity, role in cellular response to glucose starvation, hyphal growth, respons
Afu4g06950	Chr4_A_fumigatus_Af293:1,797,998..1,799,592(-)	Putative endoplasmic reticulum (ER) type II integral membrane protein with a predicted role in protein transport
Afu4g06960	Chr4_A_fumigatus_Af293:1,800,295..1,801,952(+)	Ortholog(s) have cytosol, nucleus localization
Afu4g06970	Chr4_A_fumigatus_Af293:1,801,970..1,804,581(+)	Ortholog(s) have single-stranded DNA binding activity

Afu4g06980	Chr4_A_fumigatus_Af293:1,805,075..1,808,198(-)	Ortholog of A. nidulans FGSC A4 : AN4429, A. niger CBS 513.88 : An04g01380, Neosartorya fischeri NRRL 181 : NFIA_109260, As
Afu4g06990	Chr4_A_fumigatus_Af293:1,809,133..1,811,105(-)	Ortholog(s) have endoplasmic reticulum localization
Afu4g07000	Chr4_A_fumigatus_Af293:1,813,976..1,814,860(-)	Ortholog(s) have role in actin filament organization, endocytosis and cytosol, nucleus localization
Afu4g07010	Chr4_A_fumigatus_Af293:1,815,689..1,817,552(-)	Ortholog(s) have cytosol, nucleus localization
Afu4g07020	Chr4_A_fumigatus_Af293:1,817,715..1,820,221(+)	Has domain(s) with predicted catalytic activity, heme binding, oxidoreductase activity
Afu4g07030	Chr4_A_fumigatus_Af293:1,821,787..1,823,809(+)	Transcript up-regulated in conidia exposed to neutrophils
Afu4g07040	Chr4_A_fumigatus_Af293:1,825,983..1,827,407(+)	Putative secreted aspartic-type endopeptidase
Afu4g07050	Chr4_A_fumigatus_Af293:1,827,769..1,829,194(-)	Putative lactate dehydrogenase
Afu4g07060	Chr4_A_fumigatus_Af293:1,830,102..1,831,555(+)	Ortholog of A. nidulans FGSC A4 : AN10552, A. niger CBS 513.88 : An04g01460, A. oryzae RIB40 : AO090023000870, Aspergillus
Afu4g07070	Chr4_A_fumigatus_Af293:1,831,859..1,832,638(+)	Ortholog of A. nidulans FGSC A4 : AN10554, A. oryzae RIB40 : AO090023000869, Aspergillus wentii : Aspwe1_0248748, Aspergi
Afu4g07080	Chr4_A_fumigatus_Af293:1,834,826..1,839,269(-)	Ortholog(s) have protein tyrosine phosphatase activity
Afu4g07090	Chr4_A_fumigatus_Af293:1,841,830..1,845,671(+)	Ortholog of A. nidulans FGSC A4 : AN4418, A. niger CBS 513.88 : An04g01500, A. oryzae RIB40 : AO090023000866, Aspergillus
Afu4g07100	Chr4_A_fumigatus_Af293:1,846,677..1,848,956(-)	Ortholog(s) have protein anchor activity and role in establishment of spindle pole body localization to nuclear envelope, mitoti
Afu4g07110	Chr4_A_fumigatus_Af293:1,849,132..1,850,125(+)	Ortholog(s) have SNAP receptor activity, role in Golgi to vacuole transport, vacuole inheritance and Golgi apparatus, endoplasm
Afu4g07120	Chr4_A_fumigatus_Af293:1,850,501..1,851,364(-)	Ortholog(s) have holocytochrome-c synthase activity, role in cytochrome c-heme linkage and cytosol, mitochondrial intermem
Afu4g07130	Chr4_A_fumigatus_Af293:1,851,561..1,852,972(+)	Diphosphomevalonate decarboxylase
Afu4g07140	Chr4_A_fumigatus_Af293:1,853,307..1,857,890(-)	Ortholog(s) have tRNA binding activity, role in regulation of transcription from RNA polymerase II promoter, tRNA wobble uridi
Afu4g07150	Chr4_A_fumigatus_Af293:1,858,042..1,858,554(-)	Has domain(s) with predicted cytochrome-c oxidase activity
Afu4g07160	Chr4_A_fumigatus_Af293:1,858,801..1,862,723(+)	Ortholog(s) have role in regulation of circadian rhythm and TRAMP complex localization
Afu4g07170	Chr4_A_fumigatus_Af293:1,862,823..1,864,802(+)	Ortholog(s) have polyubiquitin binding, sterol binding activity and role in anaphase-promoting complex-dependent proteasom
Afu4g07180	Chr4_A_fumigatus_Af293:1,864,856..1,866,797(-)	Ortholog of A. niger CBS 513.88 : An04g01590, A. oryzae RIB40 : AO090023000857, Aspergillus wentii : Aspwe1_0247174, Asp
Afu4g07190	Chr4_A_fumigatus_Af293:1,866,827..1,868,341(-)	Ornathine carbamoyltransferase, enzyme of the arginine biosynthesis pathway
Afu4g07200	Chr4_A_fumigatus_Af293:1,868,370..1,871,510(+)	Ortholog of A. nidulans FGSC A4 : AN4408, A. niger CBS 513.88 : An04g01600, A. oryzae RIB40 : AO090023000855, Aspergillus
Afu4g07210	Chr4_A_fumigatus_Af293:1,871,931..1,873,065(-)	Mitochondrial acetolactate synthase small subunit
Afu4g07220	Chr4_A_fumigatus_Af293:1,873,275..1,875,950(+)	Has domain(s) with predicted RNA binding activity
Afu4g07230	Chr4_A_fumigatus_Af293:1,875,953..1,878,037(-)	Has domain(s) with predicted ATP binding, ATP-dependent helicase activity, helicase activity, nucleic acid binding activity and r
Afu4g07240	Chr4_A_fumigatus_Af293:1,878,162..1,881,163(-)	Ortholog of A. nidulans FGSC A4 : AN4433, A. niger CBS 513.88 : An04g01640, A. oryzae RIB40 : AO090023000851, Aspergillus
Afu4g07250	Chr4_A_fumigatus_Af293:1,881,262..1,882,555(+)	Ortholog(s) have structural constituent of ribosome activity and mitochondrial small ribosomal subunit localization
Afu4g07260	Chr4_A_fumigatus_Af293:1,882,640..1,886,990(+)	Ortholog(s) have role in positive regulation of establishment of bipolar cell polarity regulating cell shape and cell division site, c
Afu4g07270	Chr4_A_fumigatus_Af293:1,887,031..1,890,751(-)	Putative serine carboxypeptidase
Afu4g07280	Chr4_A_fumigatus_Af293:1,891,760..1,895,289(+)	cAMP-mediated signaling protein
Afu4g07290	Chr4_A_fumigatus_Af293:1,895,297..1,896,766(+)	Ortholog(s) have Y-form DNA binding, crossed form four-way junction DNA binding, crossover junction endodeoxyribonuclease
Afu4g07300	Chr4_A_fumigatus_Af293:1,896,828..1,898,857(-)	Predicted adhesin-like protein
Afu4g07310	Chr4_A_fumigatus_Af293:1,901,441..1,902,310(+)	Ortholog(s) have ATPase activity, nicotinamide-nucleotide adenylyltransferase activity
Afu4g07320	Chr4_A_fumigatus_Af293:1,902,522..1,903,550(-)	Ortholog of Aspergillus fumigatus A1163 : AFUB_064410
Afu4g07330	Chr4_A_fumigatus_Af293:1,903,569..1,905,072(+)	Ortholog(s) have endoplasmic reticulum localization
Afu4g07340	Chr4_A_fumigatus_Af293:1,905,396..1,909,011(+)	Ortholog(s) have cytoplasm, nucleus localization
Afu4g07350	Chr4_A_fumigatus_Af293:1,909,677..1,910,880(+)	Ortholog(s) have endoplasmic reticulum localization

Afu4g07360	Chr4_A_fumigatus_Af293:1,911,526..1,914,475(-)	Putative cobalamin-independent methionine synthase
Afu4g07370	Chr4_A_fumigatus_Af293:1,914,653..1,917,944(+)	Ortholog of A. niger CBS 513.88 : An04g01760, Neosartorya fischeri NRRL 181 : NFIA_108850, Aspergillus versicolor : Aspve1_0
Afu4g07380	Chr4_A_fumigatus_Af293:1,918,233..1,920,251(-)	Ortholog(s) have role in histone exchange and NuA4 histone acetyltransferase complex, Swr1 complex localization
Afu4g07390	Chr4_A_fumigatus_Af293:1,920,338..1,921,660(+)	Ortholog(s) have Golgi apparatus, endoplasmic reticulum localization
Afu4g07400	Chr4_A_fumigatus_Af293:1,921,677..1,925,436(-)	Has domain(s) with predicted ATP binding, phosphorelay response regulator activity, phosphorelay sensor kinase activity, signa
Afu4g07410	Chr4_A_fumigatus_Af293:1,925,466..1,927,837(+)	Has domain(s) with predicted catalytic activity
Afu4g07420	Chr4_A_fumigatus_Af293:1,928,130..1,928,941(-)	Ortholog(s) have endopeptidase activator activity and role in proteasomal ubiquitin-independent protein catabolic process, pr
